# Fracture load of feldspar ceramic crowns: effects of surface treatments and aging

**DOI:** 10.1007/s00784-024-06144-w

**Published:** 2025-01-08

**Authors:** Andrea Coldea, Bogna Stawarczyk, John Meinen, Valerie Lankes, Michael V. Swain, Malgorzata Roos

**Affiliations:** 1 Department of Prosthetic Dentistry, University Hospital, LMU Munich, Munich, Germany; 2 School of Aerospace, Mechanical and Mechatronic Engineering, The University of Sydney, Sydney, Australia; 3 Epidemiology, Biostatistics and Prevention Institute, University of Zurich, Zurich, Switzerland

**Keywords:** Feldspar ceramic, Airborne particle abrasion, Etching, Fracture load

## Abstract

**Objectives:**

To compare the impact of intaglio surface treatments – airborne particle abrasion and hydrofluoric acid (HF) etching – of feldspar ceramic (FEL) crowns on the fracture load (FL) and to investigate the effects of abutment materials and artificial aging. The aim was to assess whether etching could be replaced by an alternative surface roughening method.

**Materials and methods:**

FEL crowns had their intaglio surfaces either abraded (25 µm Al_2_O_3_, 0.1 MPa), etched (HF, 60 s), or untreated and then bonded to CoCrMo- and polymer-abutments. FL was measured for non-aged and aged (1.2 million mastication cycles) specimens. Data were analyzed using, Weibull modulus, two-/one-way ANOVA with Tukey HSD-post-hoc-test, t-tests, and TOST equivalence (p < 0.05).

**Results:**

For crowns bonded to CoCrMo abutments, aging affected the FL and Weibull modulus, but pretreatment methods did not. For initial specimens, airborne abraded and etched crowns were equivalent within a 400N bound, however, for aged specimens, equivalence was inconclusive. For crowns bonded to polymer-abutments, pretreatment and aging influenced the FL. Etching decreased the initial FL by over 420N compared to airborne abraded and untreated specimens. After aging, untreated crowns’ FL decreased by 528N, while airborne abraded and etched specimens showed no aging effect.

**Conclusions:**

Airborne particle abrasion of FEL crowns’ intaglio surfaces did not negatively impact FL and was comparable to etched crowns. Conclusions regarding pretreatment methods and aging differed between CoCrMo- and polymer-abutments.

Clinical relevance.

Airborne particle abrasion may be an alternative procedure for the intaglio surface treatment of FEL crowns prior bonding.

**Supplementary Information:**

The online version contains supplementary material available at 10.1007/s00784-024-06144-w.

## Introduction

The fabrication and oral integration of feldspar ceramic prosthetic restorations, such as crowns, follow a specific procedure [1, 2]. These restorations are typically indicated for single crowns and are designed and manufactured using computer-aided design (CAD) and computer-aided manufacturing (CAM) techniques. The process involves subtractive milling of block-shaped preforms using diamond burs with water cooling [3]. After milling, the crown’ outer surfaces are polished using dental handpieces equipped with appropriate grinders and polishers [4].

Before intraoral bonding to the prepared tooth, the intaglio surface of the restoration must have sufficient roughness to achieve the necessary bond strength with the luting resin composite, enhancing mechanical interlocking [5]. The standard method for roughening feldspar ceramic restorations is extraoral hydrofluoric (HF) acid etching, which preferentially dissolves the vitreous matrix, exposing crystalline structures and creating microporosities for resin cement infiltration [6]. The resulting increase in surface roughness is a consequence of this mechanism. For dental applications, diluted HF acid with concentrations ranging from 4.9% – 10% is available in a gel-like consistency [7, 8]. The HF gel is usually applied to the restoration surface for 60 s and then rinsed off [9–11]. Despite being diluted, dental HF gels are highly aggressive and can cause skin and eye burns among practitioners and patients. Additionally, unnoticed drops of HF gels can etch porcelain surfaces, necessitating extreme caution during the etching process.

Airborne particle abrasion with alumina (Al_2_O_3_) is a potential alternative for roughening the intaglio surfaces of feldspar ceramic restorations. However, feldspar ceramics are susceptible to surface treatments, and aggressive airborne particle abrasion may reduce their damage tolerance, potentially decreasing the loading capacity of restorations during clinical performance. In contrast, restorations made from materials lacking glass phases – such as polymer-based materials, zirconia and metal alloys – are typically airborne abraded with Al_2_O_3_ particles before luting [12].

The aim of the present study was to simulate a clinically relevant test setup where the intaglio surfaces of feldspar ceramic crowns were roughened either by the standard HF-etching, experimental airborne particle abrasion, or left untreated and subsequently bonded to two different abutment materials. The abutments simulated prepared teeth: polymer abutments with a dentin-like elastic modulus of approximately 19 GPa [13], and cobalt-chrome-molybdenum (CoCrMo) abutments with an elastic modulus of approximately 240 GPa [14]. The fabrication of crowns, intaglio surface roughening, and bonding procedure corresponded to clinical practice. Additionally, simulated mastication was used as artificial aging protocol to mimic in-vitro the wear of the restoration. Both initial and aged specimens were fracture load tested, with the following hypotheses: (1) different pretreatments have no effect on the fracture load, (2) the aging process has no effect on the fracture load, (3) there is equivalence of fracture load between airborne particle abraded and etched crowns, and (4) the two different abutment materials do not impact the statistical conclusions.

## Material and methods

**Fig. 1:**
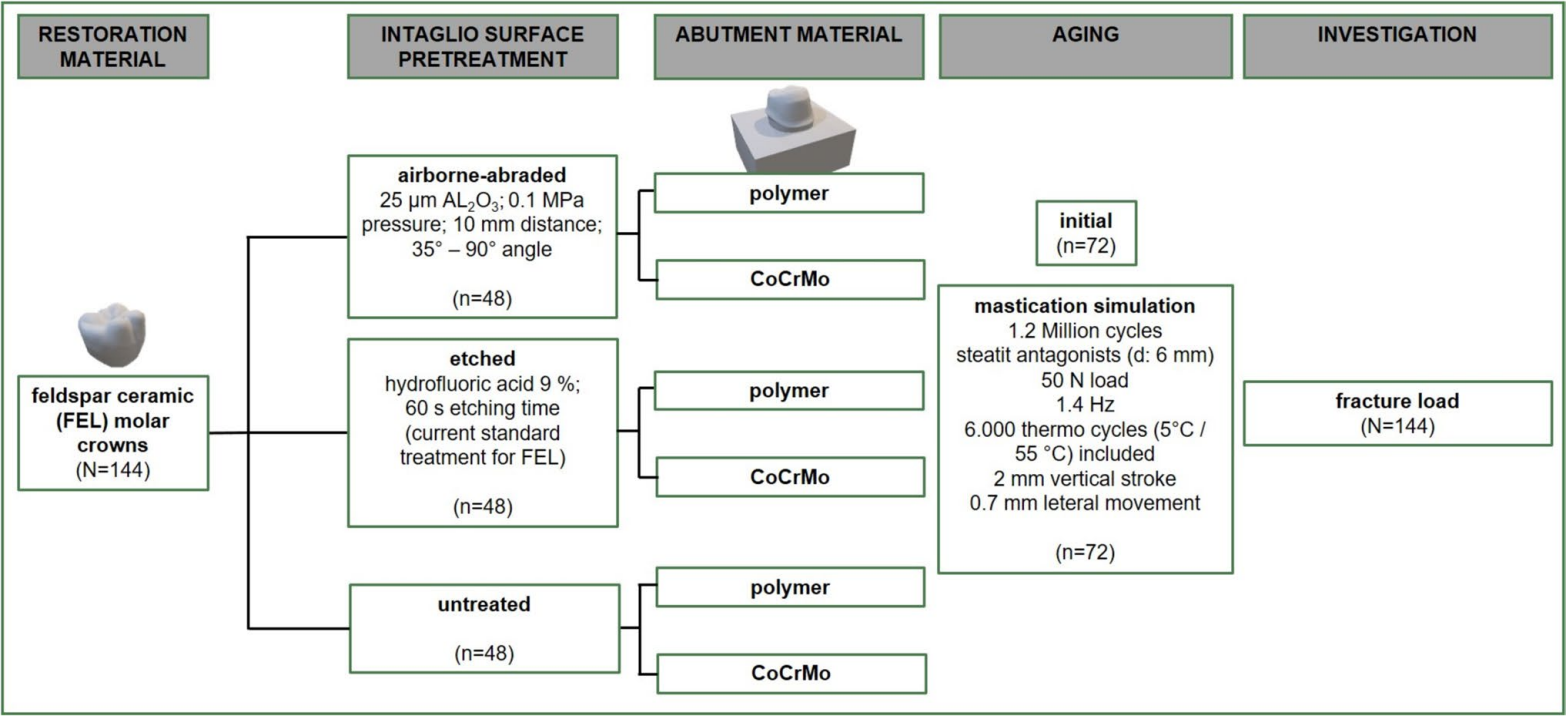
Study design

Specimens were fabricated according to the study design in Fig. 1.

### Specimen preparation

**Fig. 2:**
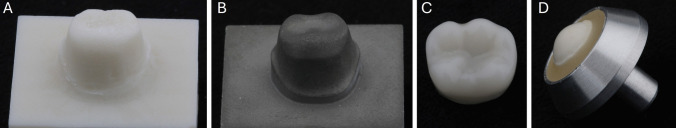
Specimen preparation; **A**: polymer abutment with dentin like E-modulus, **B**: CoCrMo abutment, **C**: feldspar ceramic crown (FEL),** D**: steatite antagonist for mastication simulation

A gypsum molar abutment was digitized employing a laboratory scanner (Ceramill Map 400, Amann Girrbach, Koblach, Austria) and its corresponding software (Ceramill Mind v2.4, Amann Girrbach, Koblach, Austria) to generate a standard tessellation language (STL) file of the abutment. On basis of the digital abutment a molar crown was designed (Ceramill Mind v2.4, Amann Girrbach). Standardized abutments were fabricated from two different CAD/CAM blank materials using the abutment STL file and a CAD/CAM milling device (Ceramill Motion 2, Amann Girrbach, Koblach, Austria). Abutments with a dentin-like modulus of elasticity of approx. 19 GPa (Fig. 2, A) were made from the polymer-based material TRINIA (TRINIA, Bicon Europe Ltd., Büchenbeuren, Germany). Cobalt-Chrome-Molybdenum (CoCrMo) metal alloy abutments (Fig. 2, B) were produced from Sintron (Ceramill Sintron, Amann Girrbach). After milling, the CoCrMo abutments were densely sintered in a furnace (Ceramill Argotherm, Amann Girrbach) under argon protective gas following the manufacturer’s instructions. A total of 144 crowns (Fig. 2, C) made from feldspar ceramic (Mark II, Vita Zahnfabrik, Bad Säckingen, Germany) were also fabricated using the same CAD/CAM milling unit (Ceramill Motion 2, Amann Girrbach). The crown surfaces were pre-polished with diamond-coated lamella rubber polishers (DIAPRO TWIST DT-H17DPmf, EVE Ernst Vetter, Keltern, Germany) and then high-gloss polished (DIAPRO TWIST DT-H17DP, EVE Ernst Vetter). The finish was performed with diamond polishing paste (9300, Gebr. Brasseler, Lemgo, Germany) and a goat hairbrush (AR9464, Gebr. Brasseler). For the aging method involving mastication simulation, 72 steatite balls (Steatite balls 1197, SD Mechatronik) were embedded in aluminum holders with epoxy resin (SCANDIQUICK, SCAN-DIA, Hagen, Germany) (Fig. 2, D).

### Crowns intaglio surface treatment and bonding

The intaglio surfaces of 48 FEL crowns were manually airborne particle abraded using an air-abrasion device (Keramo 4, Renfert, Hilzingen, Germany). A distance device maintained a 10 mm distance and the surfaces were treated for approximately 10 s at an angle of 35° to 90° using 25 µm alumina (Al_2_O_3_) particles (Cobra 25 µm, Renfert) at an air-pressure of 0.1 MPa. As a positive control group, the intaglio surfaces of another 48 FEL crowns were etched with hydrofluoric acid for 60 s (Ultradent Porcelain Etch, Ultradent, Utah, USA). Additionally, 48 FEL crowns internal surfaces were left untreated to serve as negative control group. Subsequently, all specimens were cleaned in distilled water using an ultrasonic cleaner (Transistor/Ultrasonic T-14, L&R, New Jersey, USA) for 3 min and then dried. The CoCrMo and polymer abutments were left untreated.

The self-adhesive luting resin composite RelyX Unicem Automix (3 M, Seefeld, Germany) was applied to the intaglio surfaces of the crowns using a mixing tip. The crowns were subsequently positioned onto the abutments using finger pressure, and excess material was removed. All surfaces were light-cured for 10 s each using a curing light (Elipar DeepCure-S, 3 M).

### Specimen tempering and artificial aging protocol

**Fig. 3:**
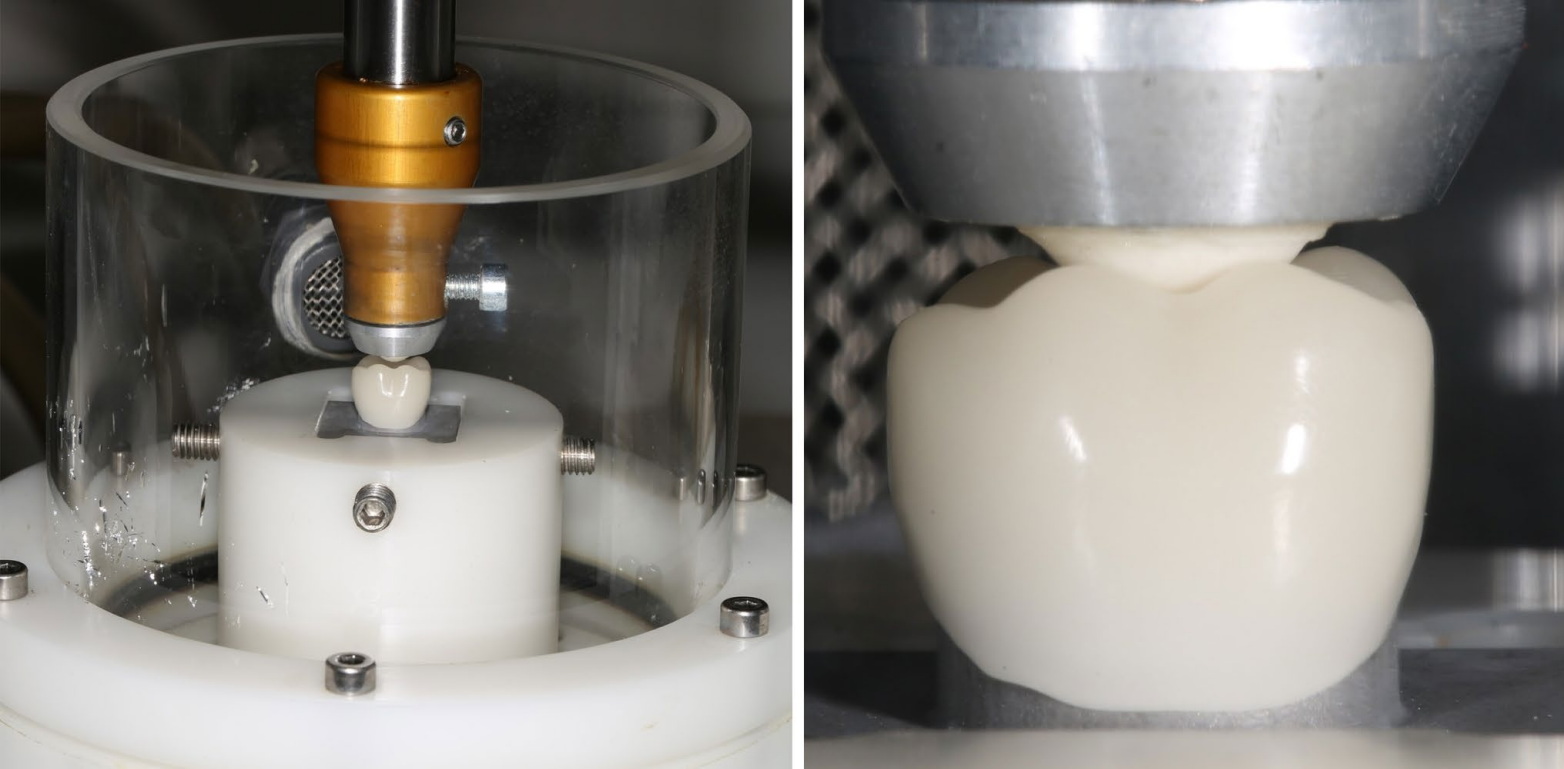
Mastication simulation set-up. Left: One representative mastication simulation chamber with specimen holder, specimen and antagonist in position. Right: close-up of antagonist and crown occlusion

After bonding crowns to abutments, the specimens were stored in 37 °C deionized water (Hera Cell 150, Heraeus, Hanau, Germany) for 24 h. According to Fig. 1, the respective groups were then artificially aged using a mastication simulator (CS-4.10, SD Mechatronik, Feldkirchen-Westerham, Germany) for 1,200,000 cycles, including approximately 6,000 thermal load cycles between 5 °C/55 °C. A load of 50 N per crown was applied using steatite antagonists at 1.4 Hz, with a vertical stroke of 2 mm and a lateral movement of 0.7 mm (Fig. 3).

### Fracture load

**Fig. 4:**
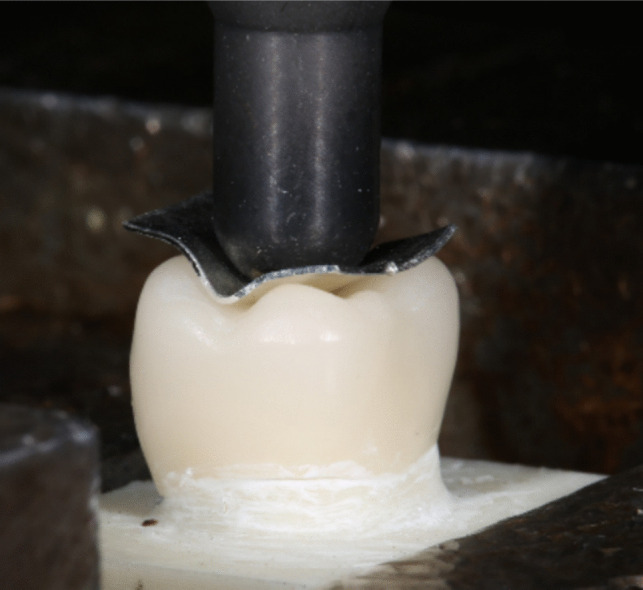
Fracture load test set-up

The fracture load of both aged and non-aged (initial) specimens was tested. The crowns bonded to the abutments were positioned in a universal testing machine (1445, Zwick Roell, Ulm, Germany) and loaded to failure, using a 6 mm diameter steel stamp with an intermediate tin foil at a crosshead speed of 1 mm/min (Fig. 4). The loading and fracture event was video recorded. The fracture load in Newton (N) was recorded. Weibull moduli and 95% confidence intervals were calculated using the maximum likelihood estimation method [15].

### Statistical analysis

Data were analyzed in R V4.3.1 (R Core Team, www.R-project.org) [16]. The normality of fracture load measurements was assessed by the Shapiro–Wilk test. Means, standard deviations, and 95% confidence intervals (95%CI) were computed separately for CoCrMo and polymer abutments. Statistical analysis included, two-way ANOVA, one-way ANOVA with Tukey HSD post-hoc test, t-tests, and TOST equivalence tests with an equivalence bound of 400 N [17, 18]. The clinically relevant equivalence bound equal to 400 N was obtained by halving the maximum mastication force of approximately 800 N in the male molar region [19]. p-values less than 0.05 were considered statistically significant.

## Results

**Fig. 5:**
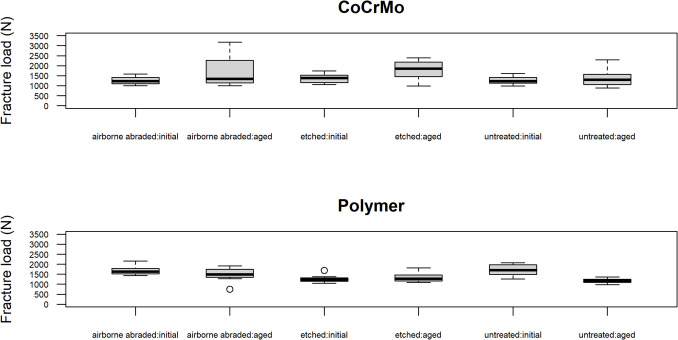
Boxplot of FEL crowns fracture loads, grouped by crowns intaglio surface pretreatment, aging and abutment material. Symbol “º” indicates extreme fracture load measurements

The fracture load measurement (n = 12) results are summarized in Fig. 5, categorized by pretreatment methods (airborne particle abraded, etched, untreated), aging status (initial, aged), and abutment material (CoCrMo and polymer).

**Fig. 6:**
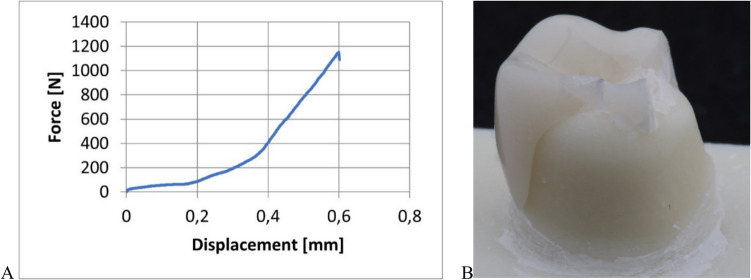
A: Typical force–displacement response for the loading to failure for a FEL crown bonded to a polymer abutment. B: Exemplary fractured FEL crown also bonded to a polymer abutment

**Fig. 7:**
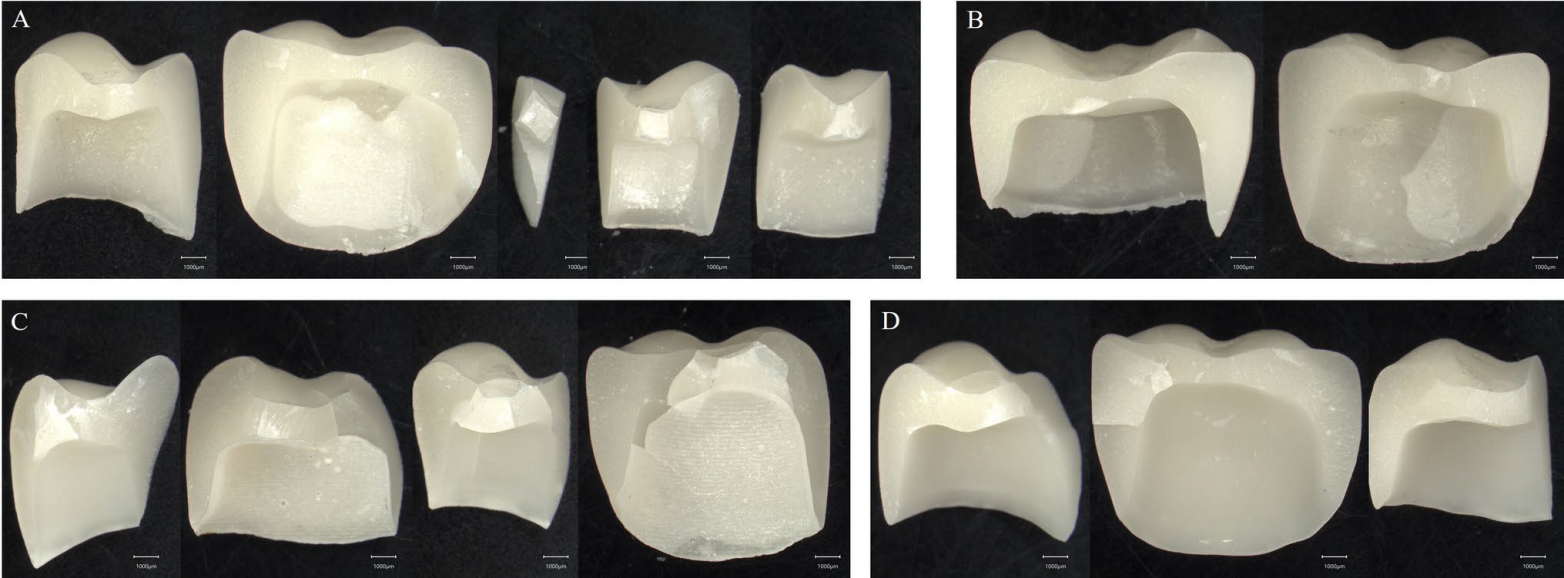
FEL crown fragments after fracture load testing. A and B: airborne abraded—bonded to CoCrMo abutments – aged, fracture load A: 2063 N (5 fragments), fracture load B: 1043 N (2 fragments). C and D: airborne abraded—bonded to polymer abutments – aged, fracture load C: 1922 N (4 major fragments), fracture load D: 1271 N (3 major fragments)

No differences were found in force–displacement responses, fracture patterns of etched, airborne abraded or untreated crowns, irrespective of abutment material. A typical force–displacement curve showing the presence of stable cracking prior to catastrophic fracture is shown in Fig. 6A, and a typical fractured crown on a polymer abutment is displayed in Fig. 6B. It was also observed, as illustrated in Fig. 7, that the number of crown fragments scaled with the fracture load value for all conditions tested. The corresponding numerical fracture load values for the CoCrMo and polymer abutments are summarized in Tables 1 and 2 respectively.

**Table 1:** Descriptive statistics of FEL crowns fracture load (in N) with 95% confidence interval of the mean and Weibull modulus with associated 95% confidence interval on CoCrMo abutments after different pretreatments and aging

abutment material	pretreatment	aging	fracture load [N] mean ± SD	fracture load [N] 95%CI	Weibull modulus (m) of fracture load	95%CI of Weibull modulus
CoCrMo	airborne-abraded	initial	1260^a^_z_ ± 195	[1136;1384]	7.9	[4.2;14.3]
		aged	1737^A^_z_ ± 779*	[1242;2232]	3	[1.5;5.4]
	etched	initial	1369^a^_y_ ± 228	[1224;1515]	6.9	[3.6;12.5]
		aged	1804^A^_z_ ± 429	[1531;2077]	4.4	[2.3;8]
	untreated	initial	1270^a^_z_ ± 195	[1146;1395]	7.7	[4.1;14]
		aged	1394^A^_z_ ± 426	[1124;1665]	4.2	[2.2;7.6]

^*a*^*: differences between pretreatments for initial specimens (one-way ANOVA)*^*A*^*: differences between pretreatments for aged specimens (one-way ANOVA)*_zy_: differences between initial and aged specimens within each pretreatment (t-test)*: Indicates the deviation from the normal distribution (Shapiro-Wilk test)

**Table 2:** Descriptive statistics of FEL crowns fracture load (in N) with 95% confidence interval of the mean and Weibull modulus with associated 95% confidence interval on polymer abutments after different pretreatments and aging

abutment material	pretreatment	aging	fracture load [N] Mean ± SD	fracture load [N] 95%CI	Weibull modulus (m) of fracture load	95%CI of Weibull modulus
polymer	airborne-abraded	initial	1675^a^_z_ ± 207	[1543;1807]	10.9	[5.9;19.8]
		aged	1504^A^_z_ ± 326	[1297;1712]	4.7	[2.4;8.6]
	etched	initial	1255^b^_z_ ± 170	[1147;1364]	9.7	[5.2;17.6]
		aged	1327^AB^_z_ ± 220	[1187;1468]	8	[4.3;14.5]
	untreated	initial	1697^a^_y_ ± 281	[1518;1876]	6.8	[3.6;12.4]
		aged	1169^B^_z_ ± 109	[1099;1239]	12.1	[6.5;22]

^*a*^*: differences between pretreatments for initial specimens (one-way ANOVA and Tukey HSD post-hoc test)*^*AB*^*: differences between pretreatments for aged specimens (one-way ANOVA and Tukey HSD post-hoc test)*

_zy_: differences between initial and aged specimens within each pretreatment (t-test)

For FEL crowns bonded to CoCrMo abutments (Fig. 5, upper graph and Table 1), when both pretreatment and aging are considered simultaneously, aging affected the fracture load (p = 0.001) while there was no evidence that pretreatment methods impacted fracture load (p = 0.121). Specifically, pretreatment had no effect on the fracture load of either initial (p = 0.371) nor aged specimens (p = 0.183). For etched specimens, aging led to an increase of fracture load by 435 N (from 1369 to 1804 N; p = 0.007). There was no evidence that aging impacted the fracture load of airborne particle abraded (p = 0.061) or untreated (p = 0.373) specimens (Table 1).

The equivalence analysis for crowns bonded to CoCrMo abutments showed that for initial specimens the fracture load of airborne particle abraded and etched crowns were within the equivalence bound of 400 N. However, for aged specimens, the impact of airborne particle abrasion and etching on fracture load was inconclusive. Weibull moduli that ranged between 3 and 7.9 (Table 1) were observed.

For FEL crowns bonded to polymer abutments (Fig. 5, lower graph and Table 2), both pretreatments (p = 0.0001) and aging (p = 0.0003) influenced the fracture load of specimens. Moreover, there was an interaction between pretreatment and aging (p = 0.0001). For initial specimens, pretreatment impacted the fracture load (p < 0.0001), with etching decreasing the fracture load by more than 420 N compared to airborne abraded and untreated specimens. For aged specimens, the fracture load is affected by pretreatment (p = 0.006), specially, untreated specimens exhibited a fracture load that was 335 N lower than that of airborne abraded specimens (p = 0.004). There was evidence that aging reduced the fracture load of untreated specimens by 528 N (p < 0.0001), while aging had no impact on the fracture load of airborne abraded and etched specimens (p > 0.143) (Table 2).

For crowns bonded to polymer abutments, there was no equivalence of FEL of initial specimens, because etching decreased the fracture load by 420 N compared to airborne abraded specimens. However, for aged specimens, the fracture load of airborne abraded and etched specimens was within the equivalence bound of 400 N. Weibull moduli ranged between 4.7 and 12.1 (Table 2).

The statistical findings for fracture loads of feldspar ceramic crowns bonded to CoCrMo and polymer abutments reported above differ greatly. Therefore, the statistical conclusions regarding the impact of pretreatments and aging on the fracture load as well as the equivalence between airborne particle abrasion and etching, strongly depend on the abutment material used.

## Discussion

Two intaglio surface treatments for feldspar ceramic were compared in vitro. Moreover, the impact of airborne particle abrasion and hydrofluoric acid etching procedures on the fracture load of molar crowns was evaluated. While the handling of hydrofluoric acid can be hazardous, airborne particle abrasion is a safer procedure. Airborne particle abrasion with alumina particles of varying grain sizes and air-pressure is the standard pre-bonding roughening procedure of polymer, alloy and zirconia restorations as reported in previous studies [20–24]. However, no prior study has analyzed the impact of airborne particle abrasion compared to the standard hydrofluoric acid etching on feldspar ceramic crowns.

In this study, a clinically relevant setup was used for the manufacturing and pretreatment process to simulate oral conditions. The intaglio surfaces of CAD/CAM-fabricated feldspar ceramic crowns were roughened either by airborne particle abrasion, hydrofluoric acid etching while control groups were left untreated after CAD/CAM machining. While the etching procedure corresponds to the standard roughening procedure used for feldspar restorations, the airborne particle abrasion parameters were chosen on the basis of zirconia roughening parameters and pre-investigations for this study. Polymers and alloys are not susceptible to particle abrasion induced damages but exhibit material ablation and are typically airborne abraded with 50 µm at 0.1 MPa [22] and with 50 – 110 µm at maximum 0.6 MPa [25] alumina particles size and air pressure respectively. In contrast, zirconia is prone to flaw introduction upon airborne abrasion and alumina particle sizes of 50 µm with maximum 0.25 MPa air pressure should be used [26]. A pre-investigation (to be published) showed a reduction in flexural strength of feldspar ceramic bending bars when airborne abraded with 50 µm and 0.1 MPa. Therefore, in the present study, the alumina particle size was reduced to 25 µm with an air pressure of 0.1 MPa. In addition to the micromechanical treatment, to enhance the bond strength, feldspathic ceramic should be chemically treated with a silane coupling agent prior bonding with a resin cement. To simulate a worst-case scenario, the silane application to the crown intaglio surfaces was omitted but should be included in future studies. Furthermore, the primary focus of the present study was on the fracture load rather than the bonding mechanisms. This limitation of the study should be addressed in future studies to analyze the bonding mechanisms in more detail.

The crowns were bonded either to fiber-reinforced polymer abutments with dentin-like elastic modulus of 18.8 GPa [13] or to CoCrMo abutments with an elastic modulus of approximately 240 GPa [14], and then artificially aged in a mastication simulator before being loaded until fracture. For mastication simulation, the applied load was standardized and consistent for all specimens and the employed equipment specially designed to emulate the oral environment. The load of 50 N applied in the mastication simulator aligns with standard practices for simulating physiological mastication forces [27]. Repeated cyclic loading in the mastication simulator can lead to the initiation and propagation of microcracks (which might be induced during airborne particle abrasion). Depending on intrinsic flaw sizes or/and possible microcracks, even low cyclic forces can induce material fatigue over time due to the repetitive nature of the load. Although the stresses generated by low simulated mastication loads remain below the material’s characteristic strength, they can still facilitate subcritical crack growth, leading to strength degradation and potential catastrophic failure [28].

A steatite sphere was chosen as the antagonist due to its standardized mechanical and physical properties, allowing for reproducible and controlled testing conditions. The spherical antagonist delivers consistent contact points and forces. This controlled setup is commonly employed in laboratory simulations aimed at evaluating the mechanical response of dental materials under standardized conditions.

Dynamic loading of the crowns was combined with thermocycling, which is an established method to replicate the oral environment. It is suggested that simulated 1.2 million mastication cycles is equivalent to approximately 5 years of in vivo wear [29]. However, this estimate is primarily based on extrapolations from 4-year clinical data on amalgam fillings and 6-month data on composite inlays [30].

Based on the present results, Hypothesis (1) stating that different pretreatments have no effect on the fracture load was confirmed for crowns bonded to CoCrMo abutments but rejected for crowns bonded to polymer abutments, as initial fracture load values of etched crowns were 420 N lower than those of airborne abraded and untreated crowns. Thus, airborne particle abrasion does not negatively affect the flexural strength of feldspar crowns. Supporting this finding, an in-vitro study revealed that the shear bond strength values of luting resin composite to feldspar ceramics are similar when either airborne particle abrasion or etching is used to roughen the bonding surfaces [31]. In contrast to the results of the present study, a study [32] found that acid-etched, disc shaped feldspar ceramic specimens showed higher mechanical strength compared to airborne abraded specimens, suggesting that clinically, acid-etching of feldspar ceramic should be preferred over airborne particle abrasion with alumina. For untreated and aged crowns bonded to polymer abutments, the fracture load was 336 N lower than that of airborne abraded crowns. This may be explained by the aging process during mastication simulation, where constant forces over 1 million cycles lead to a debonding of luting material to feldspar ceramic crowns and hence a loss of bonding support.

Hypothesis (2) stating that the aging process has no effect on the fracture load was rejected because, for airborne-abraded and etched crowns bonded to CoCrMo abutments, the fracture load was 477 N and 435 N respectively higher after mastication simulation. The substantial increased strength and observed number of fragments of these air-abraded and etched crowns after aging suggests two potential mechanisms responsible for this outcome. The aging may have resulted in plasticization of the resin bonding cement thereby reducing the magnitude of the tensile stresses during loading, especially in the area of the margins, and/or of the stress concentration associated with the defects or flaws responsible for failure has reduced. It is not clear as to the origin of this phenomena and further detailed fractographic observations and numerical modelling is required to address this question in future studies. Untreated feldspar ceramic crowns bonded to polymer abutments were influenced by aging, as the flexural strength was 528 N lower compared to initial values. This effect may be attributed to the increased likelihood of debonding in untreated crowns from the polymer abutments during aging, which could have increased stresses along the marginal and interfacial regions. In contrast, the fracture load of etched and airborne abraded crowns bonded to polymer abutments was not influenced by aging. In conclusion, feldspar ceramic restorations need to be roughened prior to bonding either by etching or airborne particle abrasion to prevent debonding and thus maintain the initial loading capacity in the oral environment. This finding is also reported in a previous study, where the fracture load of bonded glass ceramic crowns was significantly higher compared to unbonded [33].

A non-significant test result does not confirm the absence of an effect. To provide robust statistical support for the lack of effect, equivalence testing is required [17]. The hypothesized equivalence between airborne abraded and etched crowns (Hypothesis 3), regarding their fracture load values, was accepted for aged crowns bonded to polymer abutments. This provides compelling statistical evidence within a clinically relevant setting that etching can be safely substituted with airborne abrasion, without compromising the fracture load of aged feldspar crowns bonded to polymer abutments. Consequently, airborne particle abrasion is a promising alternative, pre-bonding roughening procedure of feldspar crowns, to avoid the use of hydrofluoric acid gels. In contrast, for etched and airborne abraded crowns bonded to CoCrMo abutments, equivalence was present only in the initial state. Since fracture load values of above 1169 N (mean values) exceeded the maximum mastication forces (approximately 800 N peak value in the male molar region [19]) in the oral environment the clinically relevant equivalence bound was set to 400 N.

Hypothesis (4) stated that the two different abutment materials, CoCrMo and polymer, do not impact the statistical conclusions. This hypothesis was rejected, as the abutment material strongly influenced the statistical conclusions on the fracture loads and equivalence findings of airborne abraded and etched crowns. Feldspar ceramic has an E modulus (70 GPa) and lies almost midway between that of the polymer (19 GPa) and CoCrMo (240 GPa) abutment materials. As such upon loading (to fracture) the radial displacement of the abutment will be greater for the polymer than the CoCrMo material. As a consequence, shear stresses at the interface will be greater for the CoCrMo system [34]. Possible residual tensile stresses within the resin bonding system (initial state) could have resulted in easier interface debonding and lower strength of crowns bonded to CoCrMo compared to polymer abutments in the initial state.

A previous investigation examined the shear bond strength of feldspar ceramic specimens to a resin composite cement using identical surface pretreatments to the present study (airborne abrasion with 25 µm alumina and 0.1 MPa air pressure as well as hydrofluoric acid etching) with clearly roughened surfaces [35]. A shear bond strength of 18.9 MPa initial and 26.8 MPa after thermo-cycling was measured for airborne abraded specimens. Hydrofluoric surface pretreatment led to a shear bond strength of 19.8 MPa initial and 16.2 MPa after aging. With respect to the bond strength, the results of the previous study [35] imply that airborne particle abrasion may be an alternative to acid etching roughening of feldspar ceramic and are therewith congruent with the findings of the present study where comparable fracture load values (airborne-abraded, initial: 1260 N /etched, initial: 1369 N; airborne-abraded, aged: 1737 N/etched, aged: 1804 N) were measured (Table 1).

The initial airborne abraded specimens on CoCrMo abutments (Table 1) tended to have the highest Weibull modulus of 7.9, which decreased after mastication simulation to 3. Partially debonded crowns after aging by mastication simulation could have led to a greater scatter of values compared to initial ones. However, after mastication simulation, the 95% confidence intervals overlapped and Weibull modulus values for airborne abraded (3), etched (4.4) and untreated (4.2) specimens fell within the same range.

Post-hoc sample size calculations were performed to verify the appropriateness of the sample size used in the study based on the mean and standard deviation estimates provided by the study, with alpha level set at 5%. For initial specimens, the sample size of n = 12 was sufficient and resulted in a power greater than 80%, indicating secured validity of statistical conclusions. In contrast, the results for aged specimens should be solidified by larger sample sizes in future studies. Because the original data may not be shared with third parties, the R-code that generated all statistical analyses was run on simulated mock data and provided in supplementary material to ensure computational reproducibility and complete transparency.

## Conclusions

Within the limitations of this in-vitro study, it can be concluded that roughening the intaglio surfaces of feldspar ceramic crowns using airborne particle abrasion with 25 µm Al_2_O_3_ at 0.1 MPa air-pressure does not negatively impact the fracture load and is comparable to hydrofluoric acid etching. Specifically, roughening the intaglio surfaces of feldspar ceramic restorations prior to bonding increases the loading capacity.

## Supplementary Information

Below is the link to the electronic supplementary material.

Supplementary file1 (PDF 531 KB)

## Data Availability

Data should be made available upon request.
