# Supplementary material for: Fracture load of feldspar ceramic crowns: effects of surface treatments and aging
Source: Clin Oral Investig. 2025 Jan 8;29(1):51. doi: 10.1007/s00784-024-06144-w (PMC11711631; doi:10.1007/s00784-024-06144-w)
Supplement: Supplementary file 1 — Supplementary file1 (PDF 531 KB) [file 784_2024_6144_MOESM1_ESM.pdf]

Report: Mock Data Analysis for Fracture load of feldspar ceramic crowns: effects of surface treatments and aging

Statistical Methods

Data were coded in Excel, saved in a comma separated values format and analysed in R (R Core Team) version 4.3.1. The normality assumption was assessed by the Shapiro-Wilk test. Descriptive statistics such as mean, standard deviation, and 95% confidence intervals of the mean (95%CI) of the fracture load in each group were computed. To assess the impact of the abutment material (CoCrMo and Polymer) on the statistical inference, statistical analyses were conducted separately for CoCrMo and Polymer abutment materials. To assess the impact of pretreatment and aging on the fracture load measurements, the two-way ANOVA, one-way ANOVA with the Tukey Honest Significant Differences post-hoc tests and t-tests were conducted. The equivalence of air-abrasion and etching of fracture load within the equivalence bound of 400 N was assessed by the Two One-Sided Tests (TOST) Equivalence Testing provided by the t_TOST function in the TOSTER (https://CRAN.R-project.org/package=TOSTER) package (Lakens 2017). Results of statistical analyses with p-values less than 0.05 were interpreted as statistically significant. Simulated mock data and R-code ensure complete transparency of statistical analyses. Because simulated mock data instead of true data are used to demonstrate computational reproducibility in this report, estimates and p-values can slightly differ from those reported in the manuscript.

Preparatory steps

Preparatory steps for the statistical analysis.

```r
####---- Activation of packages ----####
library(TOSTER)

####---- Set the working directory ----####

# Get the working directory and if necessary set it
# getwd()
# setwd()

####---- Read data into R ----####

dat1<-read.csv("../data/simulated_mock_data.csv", sep = ",", header=T)
dat1<-data.frame(dat1) # make sure that the format is data frame

####---- Preparation of data for analysis ----####

table(dat1$pretreatment)
```

```
 1  2  3 
48 48 48 

# pretreatment
# 1 airborne abraded (experimental pre-treatment of interest)
# 2 etched (current standard pre-treatment)
# 3 untreated (no pre-treatment)

table(dat1$abutment_material)


 1  2 
72 72 

# abutment_material
# 1 CoCrMo (current standard material for abutments)
# 2 Polymer (experimental material for abutments with elasticity similar to natural human dentin)

table(dat1$aging)


 0  1 
72 72 

# aging
# 0 no (initial)
# 1 yes (chewing simulation)

# creation of a new variable for convenience
dat1$group <- dat1$abutment_material*100 + dat1$pretreatment*10 + dat1$aging
table(dat1$group)


110 111 120 121 130 131 210 211 220 221 230 231 
 12  12  12  12  12  12  12  12  12  12  12  12 

# 110 CoCrMo & airborne abraded & initial
# 111 CoCrMo & airborne abraded & chewing simulation (aged)
# 120 CoCrMo & etched & initial
# 121 CoCrMo & etched & chewing simulation (aged)
# 130 CoCrMo & untreated & initial
# 131 CoCrMo & untreated & chewing simulation (aged)
# 210 Polymer & airborne abraded & initial
# 211 Polymer & airborne abraded & chewing simulation (aged)
# 220 Polymer & etched & initial
# 221 Polymer & etched & chewing simulation (aged)
# 230 Polymer & untreated & initial
# 231 Polymer & untreated & chewing simulation (aged)

head(dat1)
```

```
  snr specimen pretreatment abutment_material aging fracture_load_N group
1   1        1            1                 1     0          989.869   110
2   2        2            1                 1     0         1088.610   110
3   3        3            1                 1     0         1083.068   110
4   4        4            1                 1     0         1175.331   110
5   5        5            1                 1     0         1263.055   110
6   6        6            1                 1     0         1113.544   110
```

```
str(dat1)
```

```
'data.frame':   144 obs. of  7 variables:
 $ snr              : int  1 2 3 4 5 6 7 8 9 10 ...
 $ specimen         : int  1 2 3 4 5 6 7 8 9 10 ...
 $ pretreatment     : int  1 1 1 1 1 1 1 1 1 1 ...
 $ abutment_material: int  1 1 1 1 1 1 1 1 1 1 ...
 $ aging            : int  0 0 0 0 0 0 0 0 0 0 ...
 $ fracture_load_N  : num  990 1089 1083 1175 1263 ...
 $ group            : num  110 110 110 110 110 110 110 110 110 110 ...
```

```
dim(dat1)
```

```
[1] 144   7
```

```
# Split data into two CoCrMo and Polymer parts
# The main focus is on the impact of pretreatment and aging on the
fracture_load_N for CoCrMo and Polymer abutments separately.
dat_CoCrMo <- dat1[dat1$abutment_material==1,]
dat_Polymer <- dat1[dat1$abutment_material==2,]
```

Boxplots

Figure Figure 1 shows boxplots of fracture load measurements (n=12) across pretreatments (airborne abraded, etched, untreated) and aging status (initial, aged) split by CoCrMo and Polymer abutments. The figures folder contains the boxplots.tiff file that was generated according to the regulations of the journal.

```
par(mfrow = c(2, 1))
par(mar = c(4, 4, 3, 0) + 0.1, mgp = c(3, 1, 0), oma = c(0.1, 0.1, 0.1, 0.1),
las = 1)

boxplot(fracture_load_N ~ group, data = dat_CoCrMo,
        xlab = " ", ylab = "Fracture load (N)", main = "CoCrMo", ylim = c(0,
3500),
        boxwex = 0.5, cex.axis = 0.6,
        at = c(1:6),
        names = c("airborne abraded:initial", "airborne abraded:aged",
"etched:initial", "etched:aged", "untreated:initial", "untreated:aged"))

boxplot(fracture_load_N ~ group, data = dat_Polymer,
        xlab = " ", ylab = "Fracture load (N)", main = "Polymer", ylim = c(0,
3500),
        boxwex = 0.5, cex.axis = 0.6,
```

```
        at = c(1:6),
        names = c("airborne abraded:initial", "airborne abraded:aged",
"etched:initial", "etched:aged", "untreated:initial", "untreated:aged"))
```

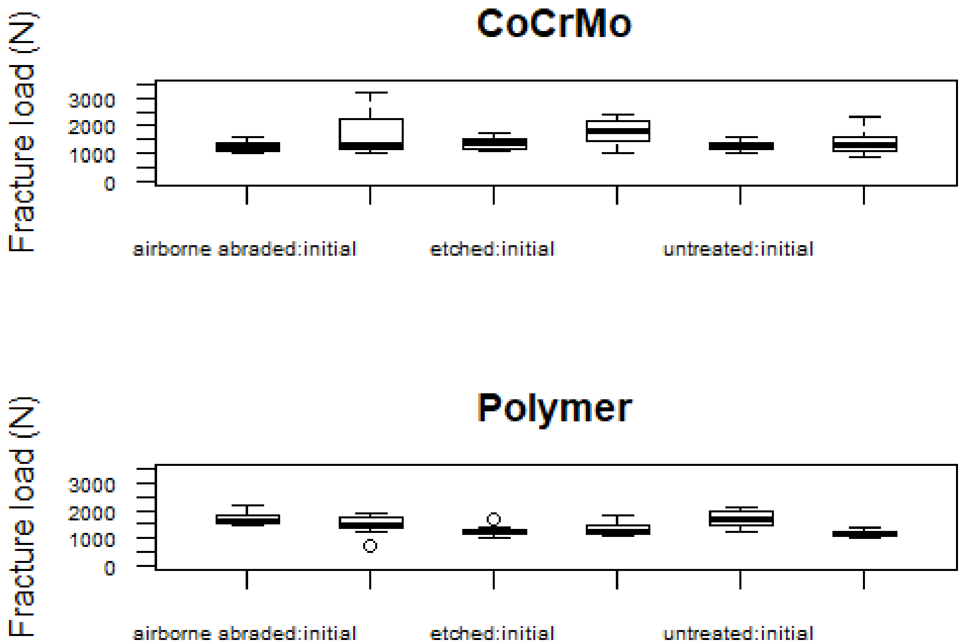

Figure 1: Boxplots of fracture load measurements (n=12) across pretreatments (airborne abraded, etched, untreated) and aging status (initial, aged) split by CoCrMo and Polymer abutments.

```
# Generation of the figure according to the regulations of the journal
# tiff format
# minimum 300 dpi
# <10MB
# black and white without shading

# 0.67 of the original
tiff(file = "../figures/boxplots.tiff", width = 2412, height = 1407,
     units = "px", res = 300)
# 9943KB

par(mfrow = c(2, 1))
par(mar = c(4, 4, 3, 0) + 0.1, mgp = c(3, 1, 0), oma = c(0.1, 0.1, 0.1, 0.1),
las = 1)

boxplot(fracture_load_N ~ group, data = dat_CoCrMo,
        xlab = " ", ylab = "Fracture load (N)", main = "CoCrMo", ylim = c(0,
3500),
        boxwex = 0.5, cex.axis = 0.6,
        at = c(1:6),
```

```
        names = c("airborne abraded:initial", "airborne abraded:aged",
"etched:initial", "etched:aged", "untreated:initial", "untreated:aged"))

boxplot(fracture_load_N ~ group, data = dat_Polymer,
        xlab = " ", ylab = "Fracture load (N)", main = "Polymer", ylim = c(0,
3500),
        boxwex = 0.5, cex.axis = 0.6,
        at = c(1:6),
        names = c("airborne abraded:initial", "airborne abraded:aged",
"etched:initial", "etched:aged", "untreated:initial", "untreated:aged"))

dev.off()

png
  2
```

Descriptive statistics

```
####---- Descriptive statistics in each group ----####

descr_stat <- function(dat){
  # This function computes descriptive statistics for measurements identified
by group
  glevels <- as.integer(dimnames(table(dat$group))[[1]])
  results <- matrix(NA, nrow=length(glevels), ncol=6)
  colnames(results)<-c("group","mean","sd","95%CILow","95%CIUp","Shapiro-Wilk
p-value")
  for(i in 1:length(glevels)){
    pos<-glevels[i]
    sample<-dat$fracture_load_N[dat$group == pos]
    results[i,1]<-pos
    results[i,2]<-round(mean(sample), 0) # mean
    results[i,3]<-round(sd(sample), 0) # SD
    results[i,4]<-floor(t.test(sample)$conf.int[1]) # 95%CILow
    results[i,5]<-ceiling(t.test(sample)$conf.int[2]) # 95%CIUp
    results[i,6]<-round(shapiro.test(sample)$p.value, 3) # Shapiro-Wilk
normality test
  }
  return(results)
}

descr_CoCrMo <- descr_stat(dat=dat_CoCrMo)
# 110 CoCrMo & airborne abraded & initial
# 111 CoCrMo & airborne abraded & chewing simulation (aged)
# 120 CoCrMo & etched & initial
# 121 CoCrMo & etched & chewing simulation (aged)
# 130 CoCrMo & untreated & initial
# 131 CoCrMo & untreated & chewing simulation (aged)
rownames(descr_CoCrMo) <- c("airborne-abraded & initial", "airborne-abraded &
aged",
                            "etched & initial", "etched & aged",
                            "untreated & initial", "untreated & aged")
```

```
descr_Polymer <- descr_stat(dat=dat_Polymer)
# 210 Polymer & airborne abraded & initial
# 211 Polymer & airborne abraded & chewing simulation (aged)
# 220 Polymer & etched & initial
# 221 Polymer & etched & chewing simulation (aged)
# 230 Polymer & untreated & initial
# 231 Polymer & untreated & chewing simulation (aged)
rownames(descr_Polymer) <- c("airborne-abraded & initial", "airborne-abraded
& aged",
                            "etched & initial", "etched & aged",
                            "untreated & initial", "untreated & aged")

knitr::kable(descr_CoCrMo)
```

Table 1: Descriptive statistics of fracture load on CoCrMo abutments.

	group	mean	sd	95%CILow	95%CIUp	Shapiro-Wilk p-value
airborne-abraded & initial	110	1260	195	1135	1384	0.299
airborne-abraded & aged	111	1738	780	1242	2235	0.027
etched & initial	120	1369	226	1225	1513	0.614
etched & aged	121	1803	430	1530	2076	0.804
untreated & initial	130	1270	196	1145	1395	0.805
untreated & aged	131	1396	425	1126	1667	0.185

```
knitr::kable(descr_Polymer)
```

Table 2: Descriptive statistics of fracture load on Polymer abutments.

	group	mean	sd	95%CILow	95%CIUp	Shapiro-Wilk p-value
airborne-abraded & initial	210	1677	207	1545	1809	0.143
airborne-abraded & aged	211	1504	327	1296	1712	0.343
etched & initial	220	1254	173	1143	1364	0.099
etched & aged	221	1327	219	1188	1467	0.112
untreated & initial	230	1699	282	1519	1879	0.406
untreated & aged	231	1169	107	1101	1238	0.998

CoCrMo: Statistical analysis

```
####---- CoCrMo analyses ----####

####---- CoCrMo: H0 (1) + H0 (2): Pretreatments and aging have no impact on
fracture load  ----####

# 2-way ANOVA with interaction for CoCrMo
aov2_CoCrMo<-aov(fracture_load_N ~ as.factor(aging) *
as.factor(pretreatment), data = dat_CoCrMo)
summary(aov2_CoCrMo)

                                         Df   Sum Sq Mean Sq F value  Pr(>F)
as.factor(aging)                          1  2159222 2159222  11.764 0.00105
**
as.factor(pretreatment)                   2   791338  395669   2.156 0.12390
as.factor(aging):as.factor(pretreatment)  2   441446  220723   1.203 0.30692
Residuals                                66 12113752  183542
---
Signif. codes:  0 '***' 0.001 '**' 0.01 '*' 0.05 '.' 0.1 ' ' 1

s_aov2_CoCrMo<-summary(aov2_CoCrMo)
```

Finding for CoCrMo: The 2-way ANOVA shows that when both pretreatment and aging are considered simultaneously, aging affects fracture load (p=0.001) and there is no evidence that pretreatments impact fracture load (p=0.124) and that there is no interaction between aging and pretreatments (p=0.307).

Now, we assess the impact of pretreatment for initial and aged specimens separately.

```
# one-way ANOVA for initial
aov1_CoCrMo_initial<-aov(fracture_load_N ~ as.factor(pretreatment), data =
dat_CoCrMo[dat_CoCrMo$aging==0, ])
summary(aov1_CoCrMo_initial)

                        Df  Sum Sq Mean Sq F value Pr(>F)
as.factor(pretreatment)  2   87156   43578   1.027  0.369
Residuals               33 1400613   42443

s_aov1_CoCrMo_initial<-summary(aov1_CoCrMo_initial)
```

Finding for CoCrMo initial: The one-way ANOVA shows that there is no impact of pretreatment on the fracture load of initial specimens (p=0.369). We do not compute post-hoc tests, because the one-way ANOVA F-test is non-significant.

```
# one-way ANOVA for aged
aov1_CoCrMo_aged<-aov(fracture_load_N ~ as.factor(pretreatment), data =
dat_CoCrMo[dat_CoCrMo$aging==1, ])
summary(aov1_CoCrMo_aged)

                        Df   Sum Sq Mean Sq F value Pr(>F)
as.factor(pretreatment)  2  1145628  572814   1.764  0.187
Residuals               33 10713139  324641
```

```
s_aov1_CoCrMo_aged<-summary(aov1_CoCrMo_aged)
```

Finding for CoCrMo aged: The one-way ANOVA shows that there is no impact of pretreatment on the fracture load of aged specimens (p=0.187). We do not compute post-hoc tests, because the one-way ANOVA F-test is non-significant.

Now, we use two-sample t-tests to assess the impact of aging for each pre-treatment separately.

```
t.test(fracture_load_N ~ aging, data=dat_CoCrMo[dat_CoCrMo$pretreatment ==
1,])


    Welch Two Sample t-test

data:  fracture_load_N by aging
t = -2.0607, df = 12.368, p-value = 0.06102
alternative hypothesis: true difference in means is not equal to 0
95 percent confidence interval:
 -982.88647   25.77577
sample estimates:
mean in group 0 mean in group 1 
       1259.759        1738.315 

t_CoCrMo_airabraded<-t.test(fracture_load_N ~ aging,
data=dat_CoCrMo[dat_CoCrMo$pretreatment == 1,])

t.test(fracture_load_N ~ aging, data=dat_CoCrMo[dat_CoCrMo$pretreatment ==
2,])


    Welch Two Sample t-test

data:  fracture_load_N by aging
t = -3.0981, df = 16.655, p-value = 0.006655
alternative hypothesis: true difference in means is not equal to 0
95 percent confidence interval:
 -730.2250 -138.0161
sample estimates:
mean in group 0 mean in group 1 
        1368.90         1803.02 

t_CoCrMo_etched<-t.test(fracture_load_N ~ aging,
data=dat_CoCrMo[dat_CoCrMo$pretreatment == 2,])

t.test(fracture_load_N ~ aging, data=dat_CoCrMo[dat_CoCrMo$pretreatment ==
3,])


    Welch Two Sample t-test

data:  fracture_load_N by aging
```

```
t = -0.93662, df = 15.466, p-value = 0.3633
alternative hypothesis: true difference in means is not equal to 0
95 percent confidence interval:
 -413.1857  160.4510
sample estimates:
mean in group 0 mean in group 1 
       1270.050        1396.417 

t_CoCrMo_untreated<-t.test(fracture_load_N ~ aging, 
data=dat_CoCrMo[dat_CoCrMo$pretreatment == 3,])
```

Finding for CoCrMo: For etched specimens, aging leads to an increase of fracture load by 1803-1369= 434N (p=0.007). There is no evidence that aging impacts the fracture load of airborne abraded (p=0.061) and untreated (p=0.363) specimens.

Now we test the equivalence of airborne abraded vs etched

```
####---- CoCrMo: H0 (3): Equivalence of airborne abraded vs etched  ----####

# Equivalence airborne abraded vs etched for initial
res_CoCrMo_initial <- t_TOST(formula = fracture_load_N ~ pretreatment,
                             data = dat_CoCrMo[(dat_CoCrMo$aging == 0) & 
(dat_CoCrMo$pretreatment<3),],
                             hypothesis = "EQU",
                             eqb = 400,
                             var.equal = FALSE,
                             smd_ci = "t")
print(res_CoCrMo_initial)


Welch Two Sample t-test

The equivalence test was significant, t(21.54) = 3.38, p < 0.01
The null hypothesis test was non-significant, t(21.54) = -1.27p = 0.22
NHST: don't reject null significance hypothesis that the effect is equal to 
zero 
TOST: reject null equivalence hypothesis

TOST Results 
                t    df p.value
t-test     -1.267 21.54   0.219
TOST Lower  3.376 21.54   0.001
TOST Upper -5.909 21.54 < 0.001

Effect Sizes 
                Estimate      SE                 C.I. Conf. Level
Raw            -109.1406 86.1676 [-257.2423, 38.9611]         0.9
Hedges's g(av)   -0.4988  0.4331    [-1.2433, 0.2456]         0.9
Note: SMD confidence intervals are an approximation. See 
vignette("SMD_calcs").

plot(res_CoCrMo_initial, type = "cd")
```

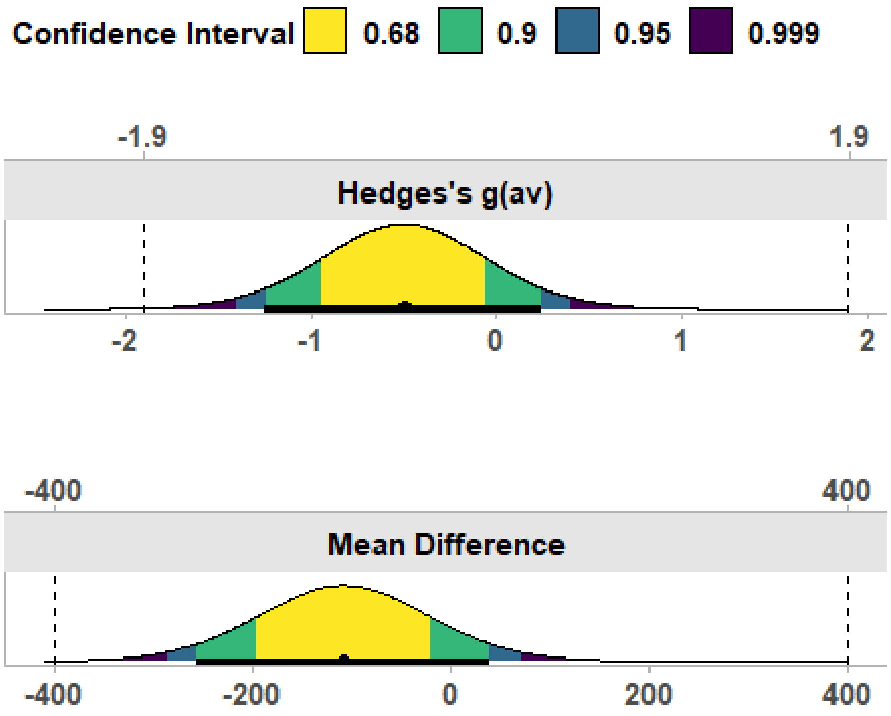


```r
plot(res_CoCrMo_initial, type = "c", ci_lines =  c(.9,.95))
```

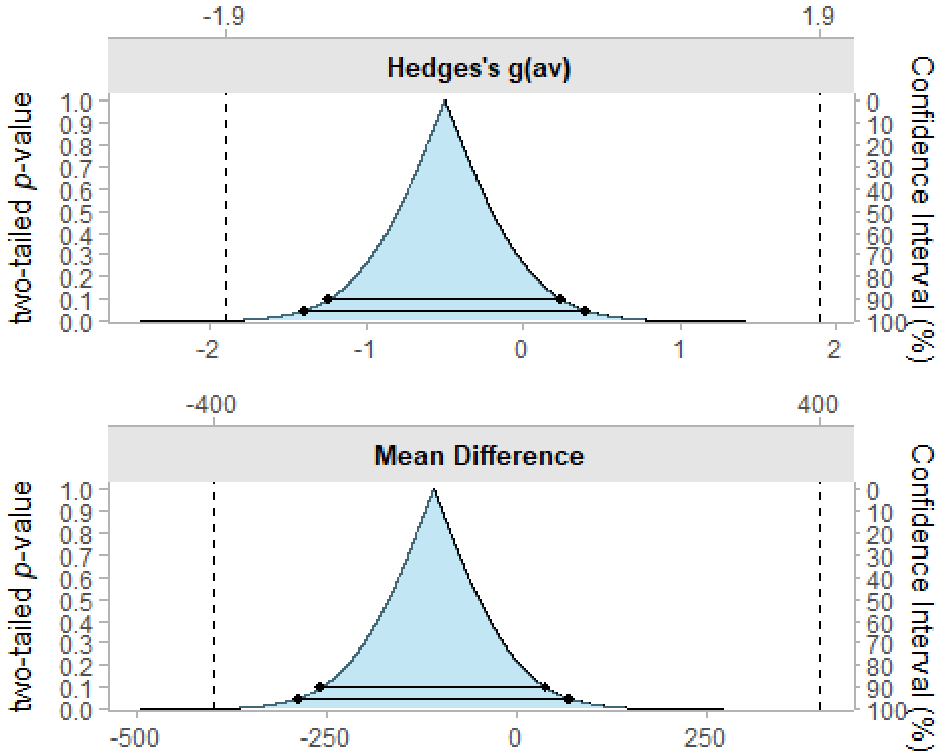


```r
describe(res_CoCrMo_initial)
```

```
[1] "Using the Welch Two Sample t-test, a null hypothesis significance test
(NHST), and a equivalence test, via two one-sided tests (TOST), were
performed with an alpha-level of 0.05. These tested the null hypotheses that
true mean difference is equal to 0 (NHST), and true mean difference is more
extreme than -400 and 400 (TOST). The equivalence test was significant,
t(21.538) = 3.376, p = 0.001 (mean difference = -109 90% C.I.[-257, 38.961];
Hedges's g(av) = -0.499 90% C.I.[-1.24, 0.246]). At the desired error rate,
it can be stated that the true mean difference is between -400 and 400."
```

Finding for CoCrMo: For initial specimens, the fracture load of airborne abraded and etched specimens is within the equivalence bound of 400N.

```
# Equivalence airborne abraded vs etched for aged
res_CoCrMo_aged <- t_TOST(formula = fracture_load_N ~ pretreatment,
                          data = dat_CoCrMo[(dat_CoCrMo$aging == 1) &
(dat_CoCrMo$pretreatment<3),],
                          hypothesis = "EQU",
                          eqb = 400,
                          var.equal = FALSE,
                          smd_ci = "t")
print(res_CoCrMo_aged)


Welch Two Sample t-test

The equivalence test was non-significant, t(17.1) = 1.3, p = 0.1
The null hypothesis test was non-significant, t(17.1) = -0.252p = 0.8
NHST: don't reject null significance hypothesis that the effect is equal to
zero
TOST: don't reject null equivalence hypothesis

TOST Results
                 t   df p.value
t-test     -0.2516 17.1   0.804
TOST Lower  1.3037 17.1   0.105
TOST Upper -1.8069 17.1   0.044

Effect Sizes
                Estimate       SE                  C.I. Conf. Level
Raw            -64.70576 257.1829 [-511.9454, 382.5339]         0.9
Hedges's g(av)  -0.09813   0.4267      [-0.8402, 0.644]         0.9
Note: SMD confidence intervals are an approximation. See
vignette("SMD_calcs").

plot(res_CoCrMo_aged, type = "cd")
```

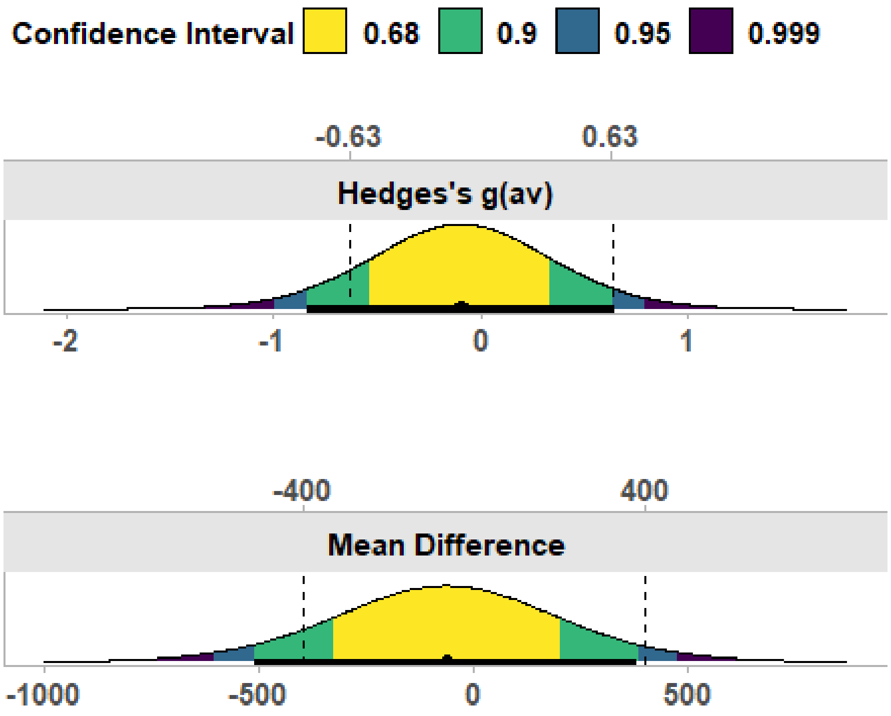


```r
plot(res_CoCrMo_aged, type = "c", ci_lines =  c(.9,.95))
```

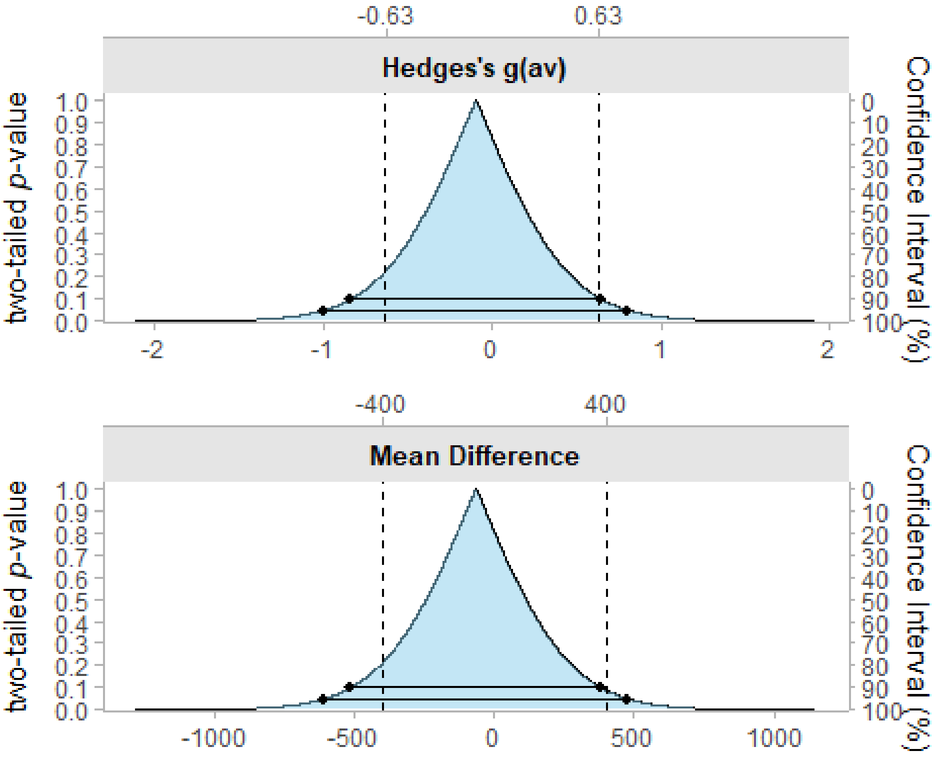


```r
describe(res_CoCrMo_aged)
```

```
[1] "Using the Welch Two Sample t-test, a null hypothesis significance test (NHST), and a equivalence test, via two one-sided tests (TOST), were performed with an alpha-level of 0.05. These tested the null hypotheses that true mean difference is equal to 0 (NHST), and true mean difference is more extreme than -400 and 400 (TOST). Both the equivalence test (p = 0.105), and the NHST (p = 0.804) were not significant (mean difference = -64.7 90% C.I.[-512, 382.534]; Hedges's g(av) = -0.0981 90% C.I.[-0.84, 0.644]). Therefore, the results are inconclusive: neither null hypothesis can be rejected."
```

Finding for CoCrMo: For aged specimens, the impact of air-abrasion and etching on the fracture load of specimens is inconclusive. There is no evidence for difference and equivalence within 400N.

Polymer: Statistical analysis

```
####---- Polymer analyses ----####

####---- Polymer: H0 (1) + H0 (2): Pretreatments and aging have no impact on fracture load  ----####

# 2-way ANOVA with interaction for Polymer
aov2_Polymer<-aov(fracture_load_N ~ as.factor(aging) * as.factor(pretreatment), data = dat_Polymer)
summary(aov2_Polymer)

                                          Df  Sum Sq Mean Sq F value   Pr(>F)
as.factor(aging)                           1  791411  791411   14.92 0.000259
as.factor(pretreatment)                    2 1081491  540746   10.19 0.000139
as.factor(aging):as.factor(pretreatment)   2 1103792  551896   10.40 0.000118
Residuals                                 66 3501574   53054

as.factor(aging)                          ***
as.factor(pretreatment)                   ***
as.factor(aging):as.factor(pretreatment) ***
Residuals
---
Signif. codes:  0 '***' 0.001 '**' 0.01 '*' 0.05 '.' 0.1 ' ' 1

s_aov2_Polymer<-summary(aov2_Polymer)
```

Finding for Polymer: The 2-way ANOVA shows that both aging (p=3^{-4}) and pretreatments (p=10^{-4}) impact the fracture load of specimens. Moreover, there is an interaction between aging and pretreatment (p=10^{-4}).

Now, we assess the impact of pretreatment for initial and aged specimens separately.

```
# one-way ANOVA for initial
aov1_Polymer_initial<-aov(fracture_load_N ~ as.factor(pretreatment), data = dat_Polymer[dat_Polymer$aging==0, ])
summary(aov1_Polymer_initial)

                        Df  Sum Sq Mean Sq F value   Pr(>F)
as.factor(pretreatment)  2 1511464  755732   14.89 2.46e-05 ***
```

```
Residuals                 33 1674664   50747
---
Signif. codes:  0 '***' 0.001 '**' 0.01 '*' 0.05 '.' 0.1 ' ' 1

s_aov1_Polymer_initial<-summary(aov1_Polymer_initial)

# Post-hoc tests
TukeyHSD(aov1_Polymer_initial)

  Tukey multiple comparisons of means
    95% family-wise confidence level

Fit: aov(formula = fracture_load_N ~ as.factor(pretreatment), data =
dat_Polymer[dat_Polymer$aging == 0, ])

$`as.factor(pretreatment)`
          diff       lwr       upr     p adj
2-1 -423.45767 -649.1254 -197.7899 0.0001703
3-1   21.60726 -204.0605  247.2750 0.9700561
3-2  445.06493  219.3972  670.7327 0.0000860

s_aov1_Polymer_initial_Tukey<-TukeyHSD(aov1_Polymer_initial)
```

Finding for Polymer: The one-way ANOVA for initial specimens shows that pretreatment impacts fracture load (p=2^{-5}). The post-hoc test shows that etching decreases the fracture load by more than 1677-1254 = 423N as compared to airborne abraded and untreated specimens (p<2^{-4}).

```
# one-way ANOVA for aged
aov1_Polymer_aged<-aov(fracture_load_N ~ as.factor(pretreatment), data =
dat_Polymer[dat_Polymer$aging==1, ])
summary(aov1_Polymer_aged)

                        Df  Sum Sq Mean Sq F value  Pr(>F)
as.factor(pretreatment)  2  673819  336910   6.086 0.00563 **
Residuals               33 1826909   55361
---
Signif. codes:  0 '***' 0.001 '**' 0.01 '*' 0.05 '.' 0.1 ' ' 1

s_aov1_Polymer_aged<-summary(aov1_Polymer_aged)

# Post-hoc tests
TukeyHSD(aov1_Polymer_aged)

  Tukey multiple comparisons of means
    95% family-wise confidence level

Fit: aov(formula = fracture_load_N ~ as.factor(pretreatment), data =
dat_Polymer[dat_Polymer$aging == 1, ])

$`as.factor(pretreatment)`
         diff       lwr       upr     p adj
```

```
2-1 -176.7622 -412.4646  58.94029 0.1724342
3-1 -334.9450 -570.6475 -99.24256 0.0039106
3-2 -158.1828 -393.8853  77.51961 0.2407873

s_aov1_Polymer_aged_Tukey<-TukeyHSD(aov1_Polymer_aged)
```

Finding for Polymer: For aged specimens, the fracture load is affected by pretreatment (p=0.006). The post-hoc tests indicate that the mean fracture load of untreated specimens is 1504-1169 = 335N lower than that of airborne abraded specimens (p=0.004).

Now, we use two-sample t-tests to assess the impact of aging for each pre-treatment separately.

```
t.test(fracture_load_N ~ aging, data=dat_Polymer[dat_Polymer$pretreatment ==
1,])


    Welch Two Sample t-test

data:  fracture_load_N by aging
t = 1.5504, df = 18.582, p-value = 0.1379
alternative hypothesis: true difference in means is not equal to 0
95 percent confidence interval:
 -60.93291 407.06261
sample estimates:
mean in group 0 mean in group 1
       1677.181        1504.116

t_Polymer_airabraded<-t.test(fracture_load_N ~ aging,
data=dat_Polymer[dat_Polymer$pretreatment == 1,])

t.test(fracture_load_N ~ aging, data=dat_Polymer[dat_Polymer$pretreatment ==
2,])


    Welch Two Sample t-test

data:  fracture_load_N by aging
t = -0.9148, df = 20.89, p-value = 0.3707
alternative hypothesis: true difference in means is not equal to 0
95 percent confidence interval:
 -241.06929   93.80797
sample estimates:
mean in group 0 mean in group 1
       1253.723        1327.354

t_Polymer_etched<-t.test(fracture_load_N ~ aging,
data=dat_Polymer[dat_Polymer$pretreatment == 2,])

t.test(fracture_load_N ~ aging, data=dat_Polymer[dat_Polymer$pretreatment ==
3,])
```

```
	Welch Two Sample t-test

data:  fracture_load_N by aging
t = 6.08, df = 14.091, p-value = 2.759e-05
alternative hypothesis: true difference in means is not equal to 0
95 percent confidence interval:
 342.9038 716.3305
sample estimates:
mean in group 0 mean in group 1 
       1698.788        1169.171 

t_Polymer_untreated<-t.test(fracture_load_N ~ aging, 
data=dat_Polymer[dat_Polymer$pretreatment == 3,])
```

Finding for Polymer: There is evidence that aging lowers the fracture load of untreated specimens by 1699-1169= 530N (p=2.8^{-5}). There is no evidence that aging impacts the fracture load of airborne abraded and etched specimens (p>0.138).

Now we test the equivalence of airborne abraded vs etched

```
####---- Polymer: H0 (3): Equivalence of airborne abraded vs etched  ----####

# Equivalence airborne abraded vs etched for initial
res_Polymer_initial <- t_TOST(formula = fracture_load_N ~ pretreatment,
                             data = dat_Polymer[(dat_Polymer$aging == 0) & 
(dat_Polymer$pretreatment<3),],
                             hypothesis = "EQU",
                             eqb = 400,
                             var.equal = FALSE,
                             smd_ci = "t")
print(res_Polymer_initial)


Welch Two Sample t-test

The equivalence test was non-significant, t(21.34) = 0.3, p = 0.62
The null hypothesis test was significant, t(21.34) = 5.444p < 0.01
NHST: reject null significance hypothesis that the effect is equal to zero 
TOST: don't reject null equivalence hypothesis

TOST Results 
                 t    df p.value
t-test      5.4438 21.34 < 0.001
TOST Lower 10.5861 21.34 < 0.001
TOST Upper  0.3016 21.34   0.617

Effect Sizes 
               Estimate     SE                 C.I. Conf. Level
Raw             423.458 77.787 [289.704, 557.2114]         0.9
Hedges's g(av)    2.143  0.538    [1.2181, 3.0683]         0.9
```

```
Note: SMD confidence intervals are an approximation. See
vignette("SMD_calcs").

plot(res_Polymer_initial, type = "cd")
```

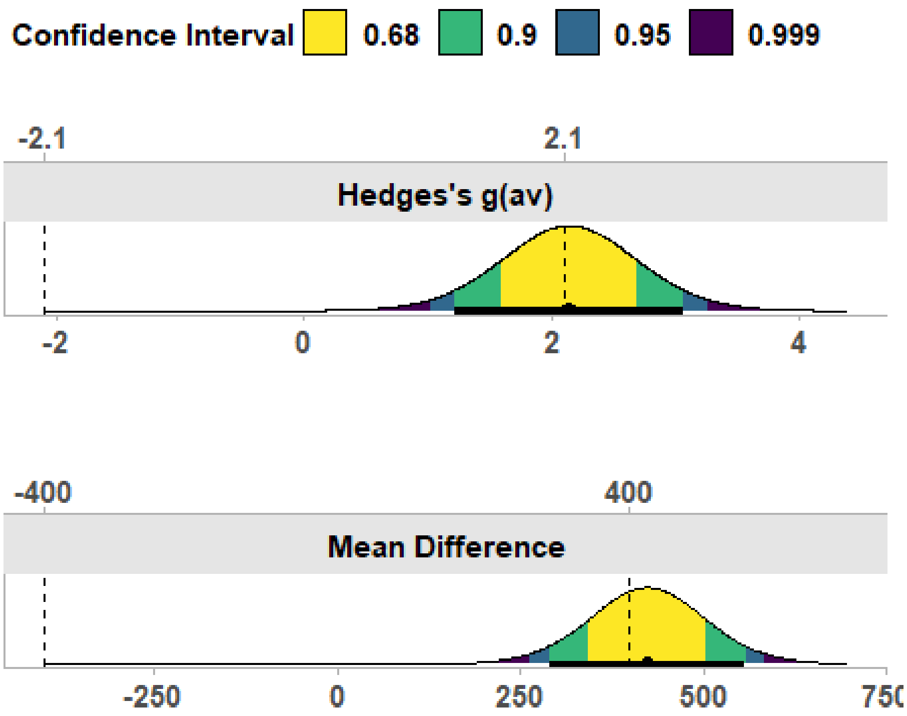


```
plot(res_Polymer_initial, type = "c", ci_lines =  c(.9,.95))
```

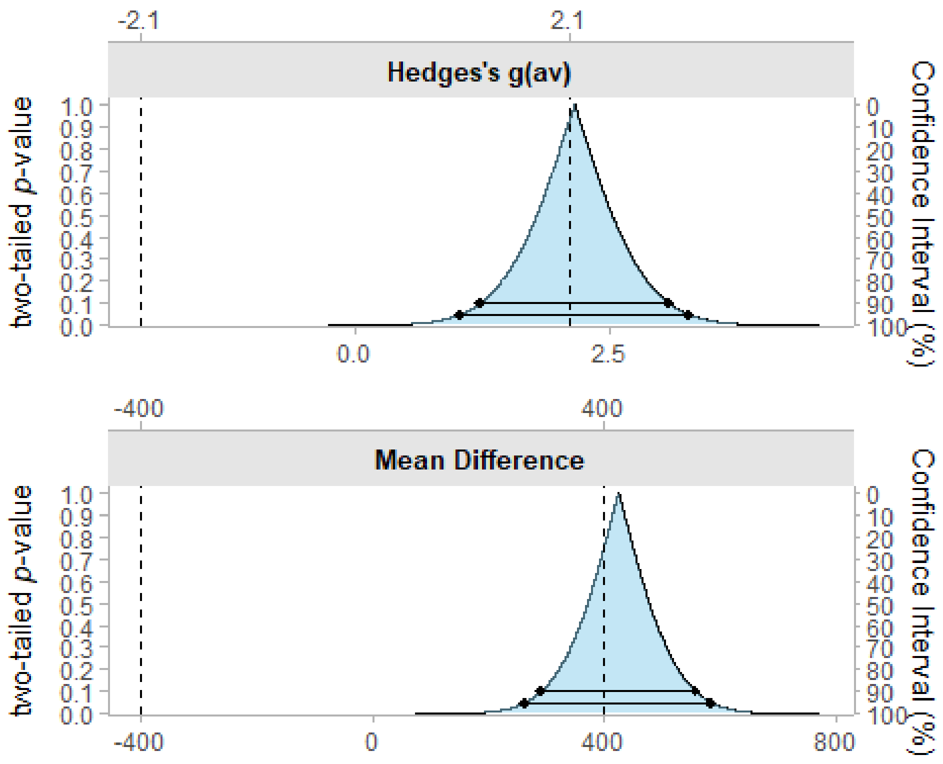


```
describe(res_Polymer_initial)
```

```
[1] "Using the Welch Two Sample t-test, a null hypothesis significance test
(NHST), and a equivalence test, via two one-sided tests (TOST), were
performed with an alpha-level of 0.05. These tested the null hypotheses that
true mean difference is equal to 0 (NHST), and true mean difference is more
extreme than -400 and 400 (TOST). The equivalence test was not significant (p
= 0.617). The NHST was significant, t(21.338) = 5.444, p < 0.001 (mean
difference = 423.458 90% C.I.[289.704, 557.211]; Hedges's g(av) = 2.143 90%
C.I.[1.218, 3.068]). At the desired error rate, it can be stated that the
true mean difference is not equal to 0 (i.e., no equivalence)."
```

Finding for Polymer: For the initial specimens, there is no equivalence because there is a difference. Etching decreases the fracture by 1677-1254 = 423N as compared to airborne abraded specimens.

```
# Equivalence airborne abraded vs etched for aged
res_Polymer_aged <- t_TOST(formula = fracture_load_N ~ pretreatment,
                           data = dat_Polymer[(dat_Polymer$aging == 1) &
(dat_Polymer$pretreatment<3),],
                           hypothesis = "EQU",
                           eqb = 400,
                           var.equal = FALSE,
                           smd_ci = "t")
print(res_Polymer_aged)


Welch Two Sample t-test
```

```
The equivalence test was significant, t(19.21) = -2, p = 0.03
The null hypothesis test was non-significant, t(19.21) = 1.557p = 0.14
NHST: don't reject null significance hypothesis that the effect is equal to
zero
TOST: reject null equivalence hypothesis

TOST Results
                 t    df p.value
t-test      1.557 19.21   0.136
TOST Lower  5.080 19.21 < 0.001
TOST Upper -1.966 19.21   0.032

Effect Sizes
                Estimate       SE                  C.I. Conf. Level
Raw             176.7622 113.5277 [-19.4351, 372.9594]         0.9
Hedges's g(av)    0.6104   0.4376   [-0.1459, 1.3667]         0.9
Note: SMD confidence intervals are an approximation. See
vignette("SMD_calcs").
```

```
plot(res_Polymer_aged, type = "cd")
```

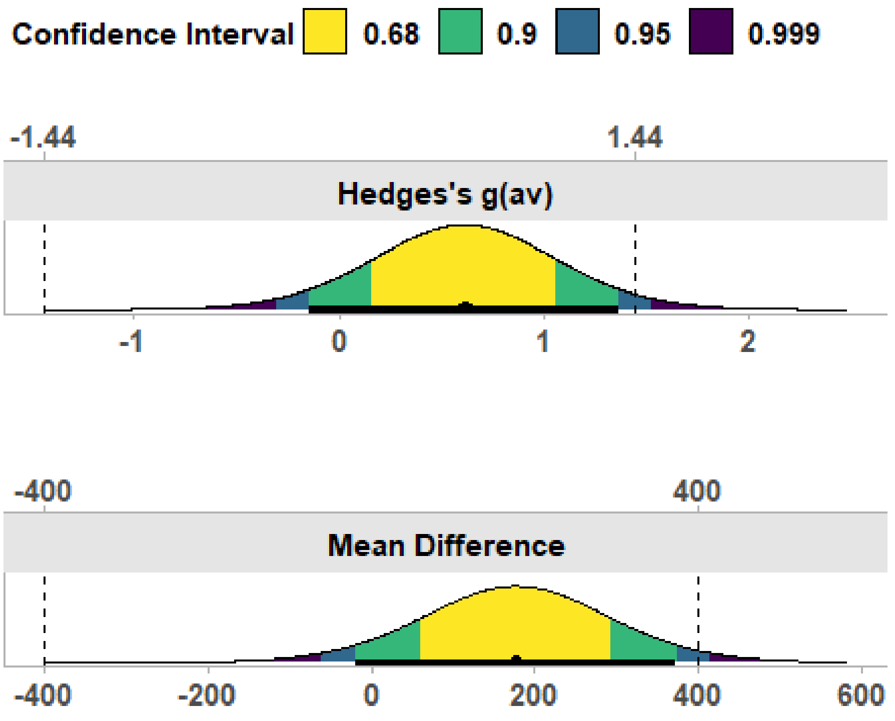


```
plot(res_Polymer_aged, type = "c", ci_lines =  c(.9,.95))
```

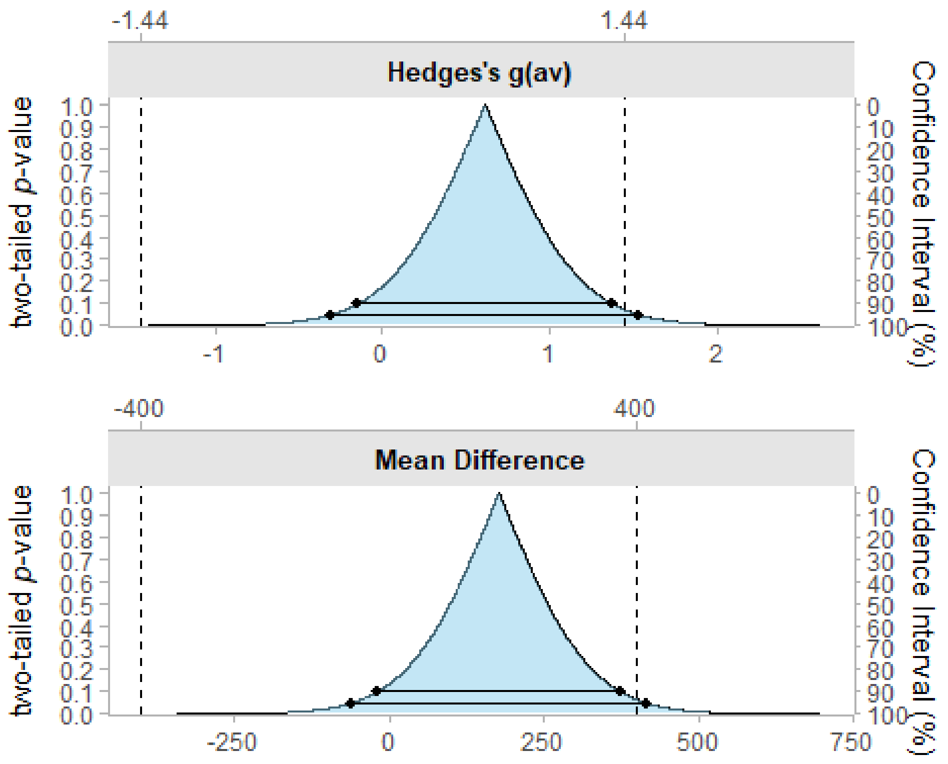


```
describe(res_Polymer_aged)

[1] "Using the Welch Two Sample t-test, a null hypothesis significance test (NHST), and a equivalence test, via two one-sided tests (TOST), were performed with an alpha-level of 0.05. These tested the null hypotheses that true mean difference is equal to 0 (NHST), and true mean difference is more extreme than -400 and 400 (TOST). The equivalence test was significant, t(19.205) = -1.97, p = 0.032 (mean difference = 176.762 90% C.I.[-19.4, 372.959]; Hedges's g(av) = 0.61 90% C.I.[-0.146, 1.367]). At the desired error rate, it can be stated that the true mean difference is between -400 and 400."
```

Finding for Polymer: For aged specimens, the fracture load of airborne abraded and etched specimens is within the equivalence bound of 400N.

```
####---- H0 (4): The two different abutment materials do not impact the fracture load inference ----####
```

Finding: Statistical inference regarding the impact of pretreatments and aging on the fracture load and the equivalence of air-abrasion and etching strongly depends on the abutment material.

References

Lakens, D. 2017. "Equivalence Tests: A Practical Primer for t Tests, Correlations, and Meta-Analyses." *Social Psychological and Personality Science* 8: 355–62.

Appendix

Sessioninfo:

```
R version 3.6.2 (2019-12-12)
Platform: x86_64-w64-mingw32/x64 (64-bit)
Running under: Windows 10 x64 (build 19045)

Matrix products: default

locale:
[1] LC_COLLATE=English_United States.1252
[2] LC_CTYPE=English_United States.1252
[3] LC_MONETARY=English_United States.1252
[4] LC_NUMERIC=C
[5] LC_TIME=English_United States.1252

attached base packages:
[1] stats     graphics  grDevices utils     datasets  methods   base

other attached packages:
[1] TOSTER_0.8.3

loaded via a namespace (and not attached):
 [1] Rcpp_1.0.3           highr_0.8            compiler_3.6.2
 [4] pillar_1.6.0         tools_3.6.2          digest_0.6.23
 [7] viridisLite_0.3.0    jsonlite_1.7.2       evaluate_0.14
[10] lifecycle_1.0.3      tibble_3.1.1         gtable_0.3.0
[13] pkgconfig_2.0.3      rlang_1.1.1          DBI_1.1.0
[16] cli_3.6.1            rstudioapi_0.14      yaml_2.2.1
[19] xfun_0.28            withr_2.5.0          stringr_1.4.0
[22] dplyr_1.0.6          knitr_1.36           generics_0.1.0
[25] vctrs_0.6.3          cowplot_1.1.1        tidyselect_1.1.0
[28] grid_3.6.2           glue_1.6.2           R6_2.4.1
[31] fansi_0.4.1          distributional_0.3.2 rmarkdown_2.11
[34] tidyr_1.1.3          purrr_0.3.4          ggplot2_3.4.2
[37] farver_2.1.0         magrittr_2.0.1       scales_1.2.1
[40] htmltools_0.4.0      ellipsis_0.3.2       ggdist_3.3.0
[43] assertthat_0.2.1     colorspace_1.4-1     labeling_0.3
[46] utf8_1.1.4           stringi_1.6.1        munsell_0.5.0
[49] crayon_1.4.1
```

Quarto version:

```
1.2.475
```

Content R-Code file

```
---
title: "Report: Mock Data Analysis for Fracture load of feldspar ceramic crowns: effects of surface treatments and aging"
bibliography: refs.bib
format:
  docx:
    fig-align: center
---

## Statistical Methods

Data were coded in Excel, saved in a comma separated values format and analysed in R (R Core Team) version 4.3.1.
The normality assumption was assessed by the Shapiro-Wilk test.
Descriptive statistics such as mean, standard deviation, and 95% confidence intervals of the mean (95%CI) of the fracture load in each group were computed.
To assess the impact of the abutment material (CoCrMo and Polymer) on the statistical inference,
statistical analyses were conducted separately for CoCrMo and Polymer abutment materials.
To assess the impact of pretreatment and aging on the fracture load measurements, the two-way ANOVA, one-way ANOVA with the Tukey Honest Significant Differences post-hoc tests and t-tests were conducted.
The equivalence of air-abrasion and etching of fracture load within the equivalence bound of 400 N was assessed by the Two One-Sided Tests (TOST) Equivalence Testing provided by the t_TOST function in the TOSTER
(https://CRAN.R-project.org/package=TOSTER) package [@lakens2017].
Results of statistical analyses with p-values less than 0.05 were interpreted as statistically significant.
Simulated mock data and R-code ensure complete transparency of statistical analyses.
Because simulated mock data instead of true data are used to demonstrate computational reproducibility in this report, estimates and p-values can slightly differ from those reported in the manuscript.

## Preparatory steps

Preparatory steps for the statistical analysis.

```{r}

####---- Activation of packages ----####
library(TOSTER)

####---- Set the working directory ----####

# Get the working directory and if necessary set it
# getwd()
# setwd()

####---- Read data into R ----####

dat1<-read.csv("../data/simulated_mock_data.csv", sep = ",", header=T)
dat1<-data.frame(dat1) # make sure that the format is data frame

####---- Preparation of data for analysis ----####

table(dat1$pretreatment)
# pretreatment
# 1         airborne abraded (experimental pre-treatment of interest)
```
```

````
# 2       etched (current standard pre-treatment)
# 3       untreated (no pre-treatment)

table(dat1$abutment_material)
# abutment_material
# 1       CoCrMo (current standard material for abutments)
# 2       Polymer (experimental material for abutments with elasticity similar to natural human dentin)

table(dat1$aging)
# aging
# 0       no (initial)
# 1       yes (chewing simulation)

# creation of a new variable for convenience
dat1$group <- dat1$abutment_material*100 + dat1$pretreatment*10 + dat1$aging
table(dat1$group)
# 110 CoCrMo & airborne abraded & initial
# 111 CoCrMo & airborne abraded & chewing simulation (aged)
# 120 CoCrMo & etched & initial
# 121 CoCrMo & etched & chewing simulation (aged)
# 130 CoCrMo & untreated & initial
# 131 CoCrMo & untreated & chewing simulation (aged)
# 210 Polymer & airborne abraded & initial
# 211 Polymer & airborne abraded & chewing simulation (aged)
# 220 Polymer & etched & initial
# 221 Polymer & etched & chewing simulation (aged)
# 230 Polymer & untreated & initial
# 231 Polymer & untreated & chewing simulation (aged)

head(dat1)
str(dat1)
dim(dat1)

# Split data into two CoCrMo and Polymer parts
# The main focus is on the impact of pretreatment and aging on the fracture_load_N for CoCrMo and Polymer
abutments separately.
dat_CoCrMo <- dat1[dat1$abutment_material==1,]
dat_Polymer <- dat1[dat1$abutment_material==2,]
```

## Boxplots

Figure @fig-boxplots shows boxplots of fracture load measurements (n=12) across pretreatments (airborne
abraded, etched, untreated) and aging status (initial, aged) split by CoCrMo and Polymer abutments. The
figures folder contains the boxplots.tiff file that was  generated according to the regulations of the journal.


```{r}
#| label: fig-boxplots
#| fig-cap: "Boxplots of fracture load measurements (n=12) across pretreatments (airborne abraded, etched,
untreated) and aging status (initial, aged) split by CoCrMo and Polymer abutments."

par(mfrow = c(2, 1))
par(mar = c(4, 4, 3, 0) + 0.1, mgp = c(3, 1, 0), oma = c(0.1, 0.1, 0.1, 0.1), las = 1)

boxplot(fracture_load_N ~ group, data = dat_CoCrMo,
````

```
    xlab = " ", ylab = "Fracture load (N)", main = "CoCrMo", ylim = c(0, 3500),
    boxwex = 0.5, cex.axis = 0.6,
    at = c(1:6),
    names = c("airborne abraded:initial", "airborne abraded:aged", "etched:initial", "etched:aged",
"untreated:initial", "untreated:aged"))

boxplot(fracture_load_N ~ group, data = dat_Polymer,
    xlab = " ", ylab = "Fracture load (N)", main = "Polymer", ylim = c(0, 3500),
    boxwex = 0.5, cex.axis = 0.6,
    at = c(1:6),
    names = c("airborne abraded:initial", "airborne abraded:aged", "etched:initial", "etched:aged",
"untreated:initial", "untreated:aged"))
```

```{r}
#| label: fig-boxplots-2-journal
#| fig-cap: "Boxplots 2"

# Generation of the figure according to the regulations of the journal
# tiff format
# minimum 300 dpi
# <10MB
# black and white without shading

# 0.67 of the original
tiff(file = "../figures/boxplots.tiff", width = 2412, height = 1407,
  units = "px", res = 300)
# 9943KB

par(mfrow = c(2, 1))
par(mar = c(4, 4, 3, 0) + 0.1, mgp = c(3, 1, 0), oma = c(0.1, 0.1, 0.1, 0.1), las = 1)

boxplot(fracture_load_N ~ group, data = dat_CoCrMo,
    xlab = " ", ylab = "Fracture load (N)", main = "CoCrMo", ylim = c(0, 3500),
    boxwex = 0.5, cex.axis = 0.6,
    at = c(1:6),
    names = c("airborne abraded:initial", "airborne abraded:aged", "etched:initial", "etched:aged",
"untreated:initial", "untreated:aged"))

boxplot(fracture_load_N ~ group, data = dat_Polymer,
    xlab = " ", ylab = "Fracture load (N)", main = "Polymer", ylim = c(0, 3500),
    boxwex = 0.5, cex.axis = 0.6,
    at = c(1:6),
    names = c("airborne abraded:initial", "airborne abraded:aged", "etched:initial", "etched:aged",
"untreated:initial", "untreated:aged"))

dev.off()
```

Descriptive statistics

```{r}
####---- Descriptive statistics in each group ----####

descr_stat <- function(dat){
  # This function computes descriptive statistics for measurements identified by group
```

```
  glevels <- as.integer(dimnames(table(dat$group))[[1]])
  results <- matrix(NA, nrow=length(glevels), ncol=6)
  colnames(results)<-c("group","mean","sd","95%CILow","95%CIUp","Shapiro-Wilk p-value")
  for(i in 1:length(glevels)){
    pos<-glevels[i]
    sample<-dat$fracture_load_N[dat$group == pos]
    results[i,1]<-pos
    results[i,2]<-round(mean(sample), 0) # mean
    results[i,3]<-round(sd(sample), 0) # SD
    results[i,4]<-floor(t.test(sample)$conf.int[1]) # 95%CILow
    results[i,5]<-ceiling(t.test(sample)$conf.int[2]) # 95%CIUp
    results[i,6]<-round(shapiro.test(sample)$p.value, 3) # Shapiro-Wilk normality test
  }
  return(results)
}

descr_CoCrMo <- descr_stat(dat=dat_CoCrMo)
# 110 CoCrMo & airborne abraded & initial
# 111 CoCrMo & airborne abraded & chewing simulation (aged)
# 120 CoCrMo & etched & initial
# 121 CoCrMo & etched & chewing simulation (aged)
# 130 CoCrMo & untreated & initial
# 131 CoCrMo & untreated & chewing simulation (aged)
rownames(descr_CoCrMo) <- c("airborne-abraded & initial", "airborne-abraded & aged",
                          "etched & initial", "etched & aged",
                          "untreated & initial", "untreated & aged")

descr_Polymer <- descr_stat(dat=dat_Polymer)
# 210 Polymer & airborne abraded & initial
# 211 Polymer & airborne abraded & chewing simulation (aged)
# 220 Polymer & etched & initial
# 221 Polymer & etched & chewing simulation (aged)
# 230 Polymer & untreated & initial
# 231 Polymer & untreated & chewing simulation (aged)
rownames(descr_Polymer) <- c("airborne-abraded & initial", "airborne-abraded & aged",
                          "etched & initial", "etched & aged",
                          "untreated & initial", "untreated & aged")
```

````
```{r}
#| label: tbl-descr_CoCrMo
#| tbl-cap: "Descriptive statistics of fracture load on CoCrMo abutments."

knitr::kable(descr_CoCrMo)
```

```{r}
#| label: tbl-descr_Polymer
#| tbl-cap: "Descriptive statistics of fracture load on Polymer abutments."

knitr::kable(descr_Polymer)
```

## CoCrMo: Statistical analysis
````

```{r}
####---- CoCrMo analyses ----####

####---- CoCrMo: H0 (1) + H0 (2): Pretreatments and aging have no impact on fracture load  ----####

# 2-way ANOVA with interaction for CoCrMo
aov2_CoCrMo<-aov(fracture_load_N ~ as.factor(aging) * as.factor(pretreatment), data = dat_CoCrMo)
summary(aov2_CoCrMo)
s_aov2_CoCrMo<-summary(aov2_CoCrMo)
```

Finding for CoCrMo: The 2-way ANOVA shows that when both pretreatment and aging are considered simultaneously, aging affects fracture load (p=`r round(s_aov2_CoCrMo[[1]][["Pr(>F)"]][1],3)`) and there is no evidence that pretreatments impact fracture load (p=`r round(s_aov2_CoCrMo[[1]][["Pr(>F)"]][2],3)`) and that there is no interaction between aging and pretreatments (p=`r round(s_aov2_CoCrMo[[1]][["Pr(>F)"]][3],3)`).

Now, we assess the impact of pretreatment for initial and aged specimens separately.

```{r}
# one-way ANOVA for initial
aov1_CoCrMo_initial<-aov(fracture_load_N ~ as.factor(pretreatment), data =
dat_CoCrMo[dat_CoCrMo$aging==0, ])
summary(aov1_CoCrMo_initial)
s_aov1_CoCrMo_initial<-summary(aov1_CoCrMo_initial)
```

Finding for CoCrMo initial: The one-way ANOVA shows that there is no impact of pretreatment on the fracture load of initial specimens (p=`r round(s_aov1_CoCrMo_initial[[1]][["Pr(>F)"]][1],3)`). We do not compute post-hoc tests, because the one-way ANOVA F-test is non-significant.

```{r}
# one-way ANOVA for aged
aov1_CoCrMo_aged<-aov(fracture_load_N ~ as.factor(pretreatment), data =
dat_CoCrMo[dat_CoCrMo$aging==1, ])
summary(aov1_CoCrMo_aged)
s_aov1_CoCrMo_aged<-summary(aov1_CoCrMo_aged)
```

Finding for CoCrMo aged: The one-way ANOVA shows that there is no impact of pretreatment on the fracture load of aged specimens (p=`r round(s_aov1_CoCrMo_aged[[1]][["Pr(>F)"]][1],3)`). We do not compute post-hoc tests, because the one-way ANOVA F-test is non-significant.

Now, we use two-sample t-tests to assess the impact of aging for each pre-treatment separately.

```{r}
t.test(fracture_load_N ~ aging, data=dat_CoCrMo[dat_CoCrMo$pretreatment == 1,])
t_CoCrMo_airabraded<-t.test(fracture_load_N ~ aging, data=dat_CoCrMo[dat_CoCrMo$pretreatment == 1,])

t.test(fracture_load_N ~ aging, data=dat_CoCrMo[dat_CoCrMo$pretreatment == 2,])
t_CoCrMo_etched<-t.test(fracture_load_N ~ aging, data=dat_CoCrMo[dat_CoCrMo$pretreatment == 2,])

t.test(fracture_load_N ~ aging, data=dat_CoCrMo[dat_CoCrMo$pretreatment == 3,])
t_CoCrMo_untreated<-t.test(fracture_load_N ~ aging, data=dat_CoCrMo[dat_CoCrMo$pretreatment == 3,])
```

````
Finding for CoCrMo: For etched specimens, aging leads to an increase of fracture load by
`r round(as.numeric(t_CoCrMo_etched$estimate[2]),0)`-`r
round(as.numeric(t_CoCrMo_etched$estimate[1]),0)`= `r round(as.numeric(t_CoCrMo_etched$estimate[2])-
as.numeric(t_CoCrMo_etched$estimate[1]),0)`N
(p=`r round(t_CoCrMo_etched$p.value,3)`).
There is no evidence that aging impacts the fracture load of airborne abraded (p=`r
round(t_CoCrMo_airabraded$p.value,3)`) and
untreated (p=`r round(t_CoCrMo_untreated$p.value,3)`) specimens.

Now we test the equivalence of airborne abraded vs etched

```{r}
####---- CoCrMo: H0 (3): Equivalence of airborne abraded vs etched  ----####

# Equivalence airborne abraded vs etched for initial
res_CoCrMo_initial <- t_TOST(formula = fracture_load_N ~ pretreatment,
                    data = dat_CoCrMo[(dat_CoCrMo$aging == 0) & (dat_CoCrMo$pretreatment<3),],
                    hypothesis = "EQU",
                    eqb = 400,
                    var.equal = FALSE,
                    smd_ci = "t")
print(res_CoCrMo_initial)
plot(res_CoCrMo_initial, type = "cd")
plot(res_CoCrMo_initial, type = "c", ci_lines =  c(.9,.95))
describe(res_CoCrMo_initial)
```

Finding for CoCrMo: For initial specimens, the fracture load of airborne abraded and etched specimens is
within the equivalence bound of 400N.

```{r}
# Equivalence airborne abraded vs etched for aged
res_CoCrMo_aged <- t_TOST(formula = fracture_load_N ~ pretreatment,
                    data = dat_CoCrMo[(dat_CoCrMo$aging == 1) & (dat_CoCrMo$pretreatment<3),],
                    hypothesis = "EQU",
                    eqb = 400,
                    var.equal = FALSE,
                    smd_ci = "t")
print(res_CoCrMo_aged)
plot(res_CoCrMo_aged, type = "cd")
plot(res_CoCrMo_aged, type = "c", ci_lines =  c(.9,.95))
describe(res_CoCrMo_aged)
```

Finding for CoCrMo: For aged specimens, the impact of air-abrasion and etching on the fracture load of
specimens is inconclusive. There is no evidence for difference and equivalence within 400N.



## Polymer: Statistical analysis

```{r}
####---- Polymer analyses ----####

####---- Polymer: H0 (1) + H0 (2): Pretreatments and aging have no impact on fracture load  ----####
````
````

````
# 2-way ANOVA with interaction for Polymer
aov2_Polymer<-aov(fracture_load_N ~ as.factor(aging) * as.factor(pretreatment), data = dat_Polymer)
summary(aov2_Polymer)
s_aov2_Polymer<-summary(aov2_Polymer)
```

Finding for Polymer: The 2-way ANOVA shows that both aging (p=`r
round(s_aov2_Polymer[[1]][["Pr(>F)"]][1],4)`) and pretreatments (p=`r
round(s_aov2_Polymer[[1]][["Pr(>F)"]][2],4)`) impact the fracture load of specimens. Moreover, there is an
interaction between aging and pretreatment (p=`r round(s_aov2_Polymer[[1]][["Pr(>F)"]][3],4)`).


Now, we assess the impact of pretreatment for initial and aged specimens separately.

```{r}
# one-way ANOVA for initial
aov1_Polymer_initial<-aov(fracture_load_N ~ as.factor(pretreatment), data =
dat_Polymer[dat_Polymer$aging==0, ])
summary(aov1_Polymer_initial)
s_aov1_Polymer_initial<-summary(aov1_Polymer_initial)

# Post-hoc tests
TukeyHSD(aov1_Polymer_initial)
s_aov1_Polymer_initial_Tukey<-TukeyHSD(aov1_Polymer_initial)
```

Finding for Polymer: The one-way ANOVA for initial specimens shows that pretreatment impacts fracture load
(p=`r round(s_aov1_Polymer_initial[[1]][["Pr(>F)"]][1],5)`). The post-hoc test shows that etching decreases
the fracture load by more than `r round(descr_Polymer[1,2],0)`-`r round(descr_Polymer[3,2],0)` = `r
round(descr_Polymer[1,2],0) - round(descr_Polymer[3,2],0)`N as compared to airborne abraded and
untreated specimens (p<`r
round(as.numeric(s_aov1_Polymer_initial_Tukey$"as.factor(pretreatment)"[1,4]),4)`).

```{r}
# one-way ANOVA for aged
aov1_Polymer_aged<-aov(fracture_load_N ~ as.factor(pretreatment), data =
dat_Polymer[dat_Polymer$aging==1, ])
summary(aov1_Polymer_aged)
s_aov1_Polymer_aged<-summary(aov1_Polymer_aged)

# Post-hoc tests
TukeyHSD(aov1_Polymer_aged)
s_aov1_Polymer_aged_Tukey<-TukeyHSD(aov1_Polymer_aged)
```

Finding for Polymer: For aged specimens, the fracture load is affected by pretreatment (p=`r
round(s_aov1_Polymer_aged[[1]][["Pr(>F)"]][1],3)`).
The post-hoc tests indicate that the mean fracture load of untreated specimens is `r
round(descr_Polymer[2,2],0)`-`r round(descr_Polymer[6,2],0)` = `r round(descr_Polymer[2,2],0) -
round(descr_Polymer[6,2],0)`N lower than that of airborne abraded specimens (p=`r
round(as.numeric(s_aov1_Polymer_aged_Tukey$"as.factor(pretreatment)"[2,4]),3)`).



Now, we use two-sample t-tests to assess the impact of aging for each pre-treatment separately.
````
````

````
```{r}
t.test(fracture_load_N ~ aging, data=dat_Polymer[dat_Polymer$pretreatment == 1,])
t_Polymer_airabraded<-t.test(fracture_load_N ~ aging, data=dat_Polymer[dat_Polymer$pretreatment == 1,])

t.test(fracture_load_N ~ aging, data=dat_Polymer[dat_Polymer$pretreatment == 2,])
t_Polymer_etched<-t.test(fracture_load_N ~ aging, data=dat_Polymer[dat_Polymer$pretreatment == 2,])

t.test(fracture_load_N ~ aging, data=dat_Polymer[dat_Polymer$pretreatment == 3,])
t_Polymer_untreated<-t.test(fracture_load_N ~ aging, data=dat_Polymer[dat_Polymer$pretreatment == 3,])
```

Finding for Polymer: There is evidence that aging lowers the fracture load of untreated specimens by `r
round(as.numeric(t_Polymer_untreated$estimate[1]),0)`-`r
round(as.numeric(t_Polymer_untreated$estimate[2]),0)`= `r
round(as.numeric(t_Polymer_untreated$estimate[1])-as.numeric(t_Polymer_untreated$estimate[2]),0)`N
(p=`r round(t_Polymer_untreated$p.value,6)`).
There is no evidence that aging impacts the fracture load of airborne abraded and etched specimens (p>`r
round(min(c(t_Polymer_airabraded$p.value, t_Polymer_etched$p.value)),3)`).

Now we test the equivalence of airborne abraded vs etched

```{r}
####---- Polymer: H0 (3): Equivalence of airborne abraded vs etched  ----####

# Equivalence airborne abraded vs etched for initial
res_Polymer_initial <- t_TOST(formula = fracture_load_N ~ pretreatment,
                       data = dat_Polymer[(dat_Polymer$aging == 0) & (dat_Polymer$pretreatment<3),],
                       hypothesis = "EQU",
                       eqb = 400,
                       var.equal = FALSE,
                       smd_ci = "t")
print(res_Polymer_initial)
plot(res_Polymer_initial, type = "cd")
plot(res_Polymer_initial, type = "c", ci_lines =  c(.9,.95))
describe(res_Polymer_initial)
```

Finding for Polymer: For the initial specimens, there is no equivalence because there is a difference.
Etching decreases the fracture by `r round(descr_Polymer[1,2],0)`-`r round(descr_Polymer[3,2],0)` = `r
round(descr_Polymer[1,2],0) - round(descr_Polymer[3,2],0)`N as compared to airborne abraded specimens.

```{r}
# Equivalence airborne abraded vs etched for aged
res_Polymer_aged <- t_TOST(formula = fracture_load_N ~ pretreatment,
                     data = dat_Polymer[(dat_Polymer$aging == 1) & (dat_Polymer$pretreatment<3),],
                     hypothesis = "EQU",
                     eqb = 400,
                     var.equal = FALSE,
                     smd_ci = "t")
print(res_Polymer_aged)
plot(res_Polymer_aged, type = "cd")
plot(res_Polymer_aged, type = "c", ci_lines =  c(.9,.95))
describe(res_Polymer_aged)
```
````

````
Finding for Polymer: For aged specimens, the fracture load of airborne abraded and etched specimens is within the equivalence bound of 400N.

```{r}
####---- H0 (4): The two different abutment materials do not impact the fracture load inference ----####
```

Finding: Statistical inference regarding the impact of pretreatments and aging on the fracture load and the equivalence of air-abrasion and etching strongly depends on the abutment material.


## References

::: {#refs}
:::

## Appendix

Sessioninfo:
```{r}
#| label: sessioninfo
#| echo: false

sessionInfo()
```

Quarto version:
```{r}
#| label: quartoversion
#| echo: false

quarto_version <- system("quarto --version", intern = TRUE)
cat(quarto_version)
```
````

Content refs.bib file

```
@article {lakens2017,
   author={Lakens, D.},
   title={Equivalence Tests: A Practical Primer for t Tests, Correlations, and Meta-Analyses},
   journal={Social Psychological and Personality Science},
   volume={8},
   year={2017},
  pages={355--362}
}
```

Content simulated_mock_data.csv file

snr	specimen	pretreatment	abutment_material	aging	fracture_load_N
1	1	1	1	0	989.869007
2	2	1	1	0	1088.6102
3	3	1	1	0	1083.06803
4	4	1	1	0	1175.33124
5	5	1	1	0	1263.05536

6	6	1	1	0	1113.54398
7	7	1	1	0	1539.7097
8	8	1	1	0	1165.90792
9	9	1	1	0	1301.00459
10	10	1	1	0	1584.22294
11	11	1	1	0	1504.533
12	12	1	1	0	1308.2544
13	1	1	2	0	1518.46772
14	2	1	2	0	2163.87978
15	3	1	2	0	1883.65016
16	4	1	2	0	1507.02139
17	5	1	2	0	1583.17959
18	6	1	2	0	1674.18436
19	7	1	2	0	1679.17911
20	8	1	2	0	1435.86603
21	9	1	2	0	1831.21297
22	10	1	2	0	1545.24789
23	11	1	2	0	1767.43095
24	12	1	2	0	1536.85305
25	1	1	1	1	1996.42098
26	2	1	1	1	2966.45823
27	3	1	1	1	2467.89283
28	4	1	1	1	3183.18596
29	5	1	1	1	2057.42015
30	6	1	1	1	1193.01997
31	7	1	1	1	1170.72883
32	8	1	1	1	1106.23694
33	9	1	1	1	1480.59545
34	10	1	1	1	1050.48414
35	11	1	1	1	1178.62252
36	12	1	1	1	1008.7086
37	1	1	2	1	1379.15076
38	2	1	2	1	1723.13287
39	3	1	2	1	1785.25351
40	4	1	2	1	1386.82232
41	5	1	2	1	1630.04773
42	6	1	2	1	1919.39337
43	7	1	2	1	1913.34947
44	8	1	2	1	748.532774
45	9	1	2	1	1445.176
46	10	1	2	1	1263.7792
47	11	1	2	1	1525.00217
48	12	1	2	1	1329.75469
49	1	2	1	0	1169.9055
50	2	2	1	0	1052.55344
51	3	2	1	0	1444.75898

52	4	2	1	0	1332.13434
53	5	2	1	0	1523.1879
54	6	2	1	0	1523.05197
55	7	2	1	0	1650.34043
56	8	2	1	0	1063.1553
57	9	2	1	0	1435.99067
58	10	2	1	0	1358.46334
59	11	2	1	0	1737.43194
60	12	2	1	0	1135.82349
61	1	2	2	0	1158.63291
62	2	2	2	0	1375.93764
63	3	2	2	0	1287.90711
64	4	2	2	0	1212.59677
65	5	2	2	0	1074.05987
66	6	2	2	0	1268.47492
67	7	2	2	0	1177.08292
68	8	2	2	0	1296.09892
69	9	2	2	0	1696.84023
70	10	2	2	0	1135.37361
71	11	2	2	0	1324.0473
72	12	2	2	0	1037.6288
73	1	2	1	1	1369.85717
74	2	2	1	1	1670.88915
75	3	2	1	1	1419.88964
76	4	2	1	1	1492.40791
77	5	2	1	1	1921.35863
78	6	2	1	1	2390.77415
79	7	2	1	1	2228.87995
80	8	2	1	1	2129.59046
81	9	2	1	1	2262.73079
82	10	2	1	1	976.620781
83	11	2	1	1	2000.54746
84	12	2	1	1	1772.69764
85	1	2	2	1	1594.07462
86	2	2	2	1	1535.34589
87	3	2	2	1	1811.37795
88	4	2	2	1	1129.93645
89	5	2	2	1	1122.89965
90	6	2	2	1	1314.19677
91	7	2	2	1	1100.05828
92	8	2	2	1	1206.62846
93	9	2	2	1	1188.94453
94	10	2	2	1	1382.74476
95	11	2	2	1	1241.84006
96	12	2	2	1	1300.20148
97	1	3	1	0	982.070732

98	2	3	1	0	1093.8536
99	3	3	1	0	1075.33669
100	4	3	1	0	1148.82028
101	5	3	1	0	1614.54298
102	6	3	1	0	1382.15495
103	7	3	1	0	1149.79975
104	8	3	1	0	1435.3624
105	9	3	1	0	1246.62772
106	10	3	1	0	1214.69591
107	11	3	1	0	1530.80329
108	12	3	1	0	1366.52739
109	1	3	2	0	1343.34176
110	2	3	2	0	1262.71373
111	3	3	2	0	1917.9031
112	4	3	2	0	1577.59475
113	5	3	2	0	1732.49705
114	6	3	2	0	1585.29079
115	7	3	2	0	1684.95099
116	8	3	2	0	1387.66221
117	9	3	2	0	1723.43655
118	10	3	2	0	2034.02638
119	11	3	2	0	2061.39222
120	12	3	2	0	2074.65055
121	1	3	1	1	1019.13819
122	2	3	1	1	1309.93079
123	3	3	1	1	1048.47111
124	4	3	1	1	1530.59063
125	5	3	1	1	1267.34678
126	6	3	1	1	2038.71989
127	7	3	1	1	1616.26569
128	8	3	1	1	1469.99287
129	9	3	1	1	1185.71786
130	10	3	1	1	1076.46951
131	11	3	1	1	2298.26451
132	12	3	1	1	896.095952
133	1	3	2	1	1096.60619
134	2	3	2	1	984.817121
135	3	3	2	1	1349.74374
136	4	3	2	1	1158.75141
137	5	3	2	1	1045.3275
138	6	3	2	1	1177.54422
139	7	3	2	1	1305.03631
140	8	3	2	1	1213.56806
141	9	3	2	1	1274.4119
142	10	3	2	1	1120.84275
143	11	3	2	1	1108.1431

144	12	3	2	1	1195.2625

Content README.txt file

Reproducible report of the Fracture Load analysis of mock data for

Coldea A, Stawarczyk B, Meinen J, Lankes V, Swain MV, Roos M. Fracture load of feldspar ceramic crowns: effects of surface treatments and aging. Clin Oral Investig. 2025 Jan 8;29(1):51. doi: 10.1007/s00784-024-06144-w.

To run the code
1. Install R (https://www.r-project.org/) on your computer
2. Install RStudio (https://posit.co/download/rstudio-desktop/) on your computer
3. Open the mock_data_report.qmd file in RStudio
4. Install TOSTER package: install.packages("TOSTER")
5. Make sure that Word program is not active on your computer
6. Klick on "Render" in RStudio
